# Assembly of the durian chloroplast genome using long PacBio reads

**DOI:** 10.1038/s41598-020-73549-4

**Published:** 2020-10-07

**Authors:** Jeremy R. Shearman, Chutima Sonthirod, Chaiwat Naktang, Duangjai Sangsrakru, Thippawan Yoocha, Ratchanee Chatbanyong, Siriporn Vorakuldumrongchai, Orwintinee Chusri, Sithichoke Tangphatsornruang, Wirulda Pootakham

**Affiliations:** 1 National Omics Center, National Science and Technology Development Agency, 111 Thailand Science Park, Paholyothin Road, Khlong Nueng, Khlong Luang, Pathumthani 12120 Thailand; 2 Department of Agriculture, Chantaburi Horticultural Research Center, Chanthaburi, 22110 Thailand

**Keywords:** Genome, Genomics, Plant genetics, Sequencing

## Abstract

We have assembled the complete sequence of the *Durio zibethinus* chloroplast genome using long PacBio reads. Durian is a valuable commercial tree that produces durian fruit, which is popular in Southeast Asia. The chloroplast genome assembled into a single 143 kb cyclic contig that contained 111 genes. There were 46 short direct repeats (45 to 586 bp) and five short inverted repeats (63 to 169 bp). The long reads that were used for the assembly span the entire chloroplast with > 10 kb overlaps and multiple long reads join the start of the contig to the end of the contig. The durian chloroplast was found to lack the large inverted repeat that is common in chloroplast genomes. An additional 24 durian varieties were sequenced and compared to the assembly and found to also lack the large inverted repeat. There were nine SNPs among the varieties.

## Introduction

Durian (*Durio zibethinus*) is a flowering tree from the family Malvaceae. It produces a large, spiky fruit with a strong husk and pungent edible flesh. The fragrance can be so pungent that the fruit is often banned from indoor public spaces. Durian is referred to as the ‘king of fruit’ and is commonly grown in Southeast Asian countries, such as Thailand, Indonesia and Malaysia. Fruit production is seasonal and the price is quite high relative to other fruits, making durian a valuable crop species. One of the most popular varieties in Thailand is Monthong, which has a large fruit, relatively mild odour and soft creamy flesh that is on the sweeter side of durian varieties.

A high-quality draft assembly of the durian genome was published in 2017 using PacBio reads and Chicago Hi-C, where approximately 95% of the ~ 738 Mb genome was covered by 30 scaffolds^1^. The durian chloroplast genome was assembled from Illumina sequence data into a 164 kb cyclic sequence^2^. Chloroplast sequences often, but not always, have a quadripartite structure consisting of a large single copy sequence and a short single copy sequence separated by a pair of large inverted repeats (IR) that range in size between 10 and 30 kb depending on the species^3^. The durian chloroplast was reported to have a large single copy sequence that was 95.7 kb and a short single copy sequence of 20.9 kb with a 23.6 kb inverted repeat^2^.

It is generally accepted that the chloroplasts, found in all members of the plant kingdom, are derived from a single ancestral origin. This is reflected in both the chloroplast genome size and organisation with high levels of synteny, among several other similarities (for review see^4^). The chloroplast genome in most species is highly conserved, which has led some to consider a functional role for the IR, such as the initiation of replication^5^, gene conservation^6,7^, or to help stabilise the genome^6^. However, some species have experienced IR expansion or contraction^8–10^ and some have lost an entire copy of the IR^6,11^ with no apparent negative consequences to the plant, which means that any function the IR may have is not required. We used long PacBio reads to assemble the durian chloroplast genome.

## Results and discussion

### Durian chloroplast assembly and annotation

**Figure 1:**
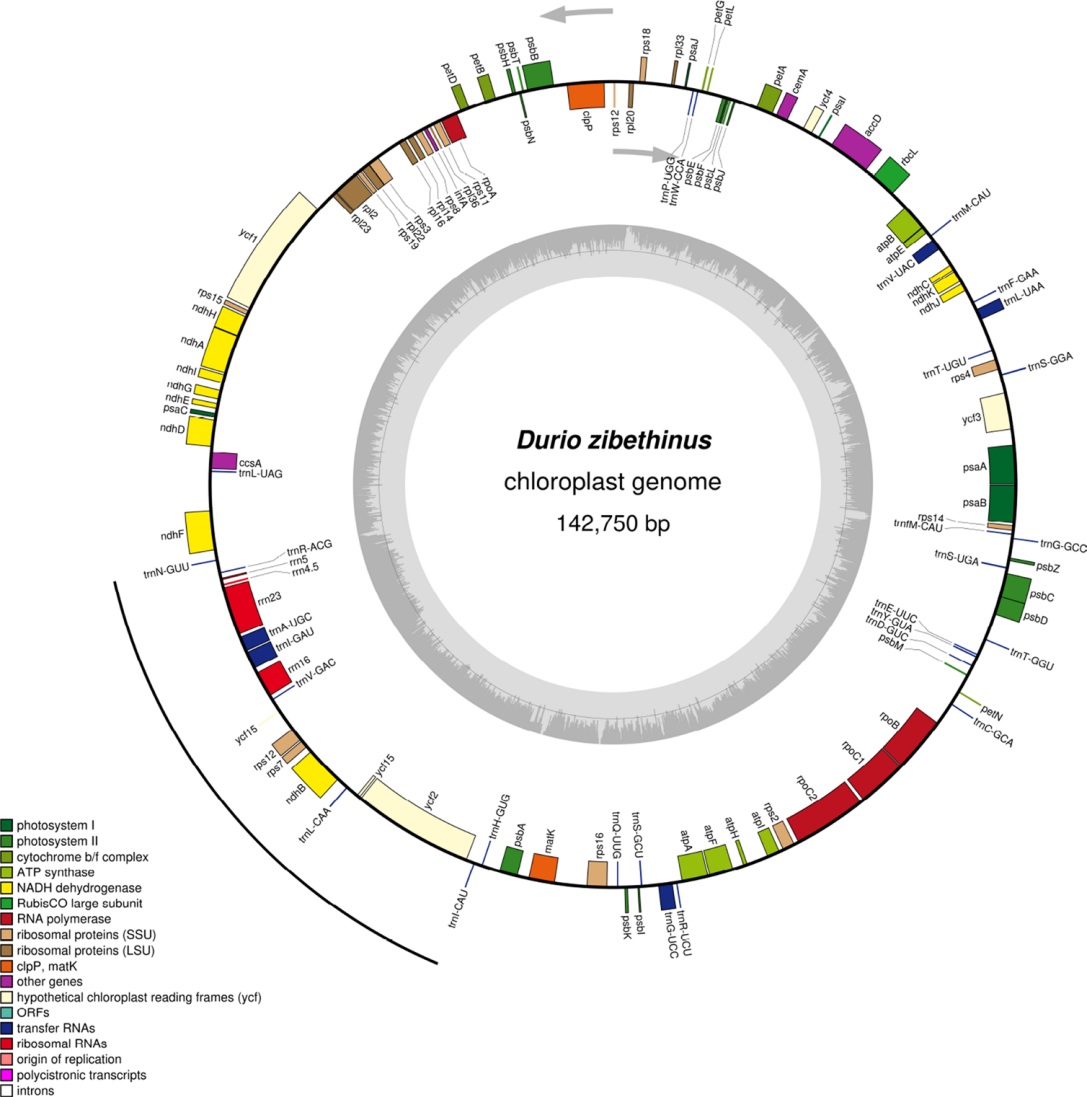
Structure of the Durio zibethinus chloroplast genome showing gene location and exon structure. Gray arrows at the top indicate transcription direction and gene location on the plus or minus strand is indicated by the exon being outside or inside the circle, respectively. GC content is indicated as a histogram on the inner circle. The sequence that typically comprises the IR is marked using the black line.

We have assembled the durian chloroplast from the Monthong variety using long PacBio reads and the CANU assembly program^12^ (with ABruijn assembler^13^ also returning the same assembly structure). The chloroplast assembled into a 142,733 bp cyclic contig that contained 111 genes (Fig. 1, Table 1). There were 46 direct repeats ranging in size from 45 to 586 bp and 5 small inverted repeats ranging in size from 63 to 169 bp. The majority of repeats were imperfect and contained several mismatches or gaps. The most striking finding was an absence of the IR that is common in plant chloroplasts. The assembled durian chloroplast contains only a single copy of the sequence that typically comprises the IR (Fig. 1). This was unexpected since the published durian chloroplast genome (MG138151.1), also from a Thai Monthong variety, was 163,974 bp and included an IR^2^. The junction of the IR in the published chloroplast is a 169 bp small inverted repeat in our assembly. This repeat is slightly longer than the short reads that were used for the published chloroplast assembly, so it is likely that this repeat caused an assembly error.

**Table 1:** Genes encoded on the *Durio zibethinus* chloroplast genome, grouped according to function.

Category	Gene groups	Name of genes
Self-replication	Large subunit of ribosomal proteins	rpl2, rpl14, rpl16, rpl20, rpl232, rpl32, rpl33, rpl36
	Small subunit of ribosomal proteins	rps2, rps3, rps4, rps7, rps8, rps11, rps12, rps14, rps15, rps16, rps18, rps19
	DNA-dependent RNA polymerase	rpoA, rpoB, rpoC1, rpoC2
	Ribosomal RNA genes	rrn4.5, rrn5, rrn16, rrn23
	Transfer RNA genes	trnA-UGC, trnC-GCA, trnD-GUC, trnE-UUC, trnF-GAA, trnfM-CAU, trnG-GCC, trnG-UCC, trnH-GUG, trnI-CAU, trnI-GAU, trnL-CAA, trnL-UAG, trnL-UAA, trnM-CAU, trnN-GUU, trnP-UGG, trnQ-UUG, trnR-ACG, trnR-UCU, trnS-GCU, trnS-UGA, trnS-GGA, trnT-UGU, trnT-GGU, trnV-GAC, trnV-UAC, trnW-CCA, trnY-GUA
Photosynthesis	Photosystem I	psaA, psaB, psaC, psaI, psaJ
	Photosystem II	psbA, psbB, psbC, psbD, psbE, psbF, psbH, psbI, psbJ, psbK, psbL, psbM, psbN, psbT, psbZ
NADH dehydrogenase	NADH dehydrogenase	ndhA, ndhB, ndhC, ndhD, ndhE, ndhF, ndhG, ndhH, ndhI, ndhJ, ndhK
	Cytochrome b/f complex	petA, petB, petD, petG, petL, petN
	ATP synthase	atpA, atpB, atpE, atpF, atpH, atpI
	RubisCo large subunit	rbcL
Other genes	Maturase K	matK
	Envelope membrane protein	cemA
	Subunit of acetyl-CoAcarboxylase	accD
	C-type cytochrome synthesis gene	ccsA
	Protease	clpP1
	Conserved hypothetical chloroplast open reading frames	ycf1, ycf2, ycf3, ycf4, ycf15

**Figure 2:**
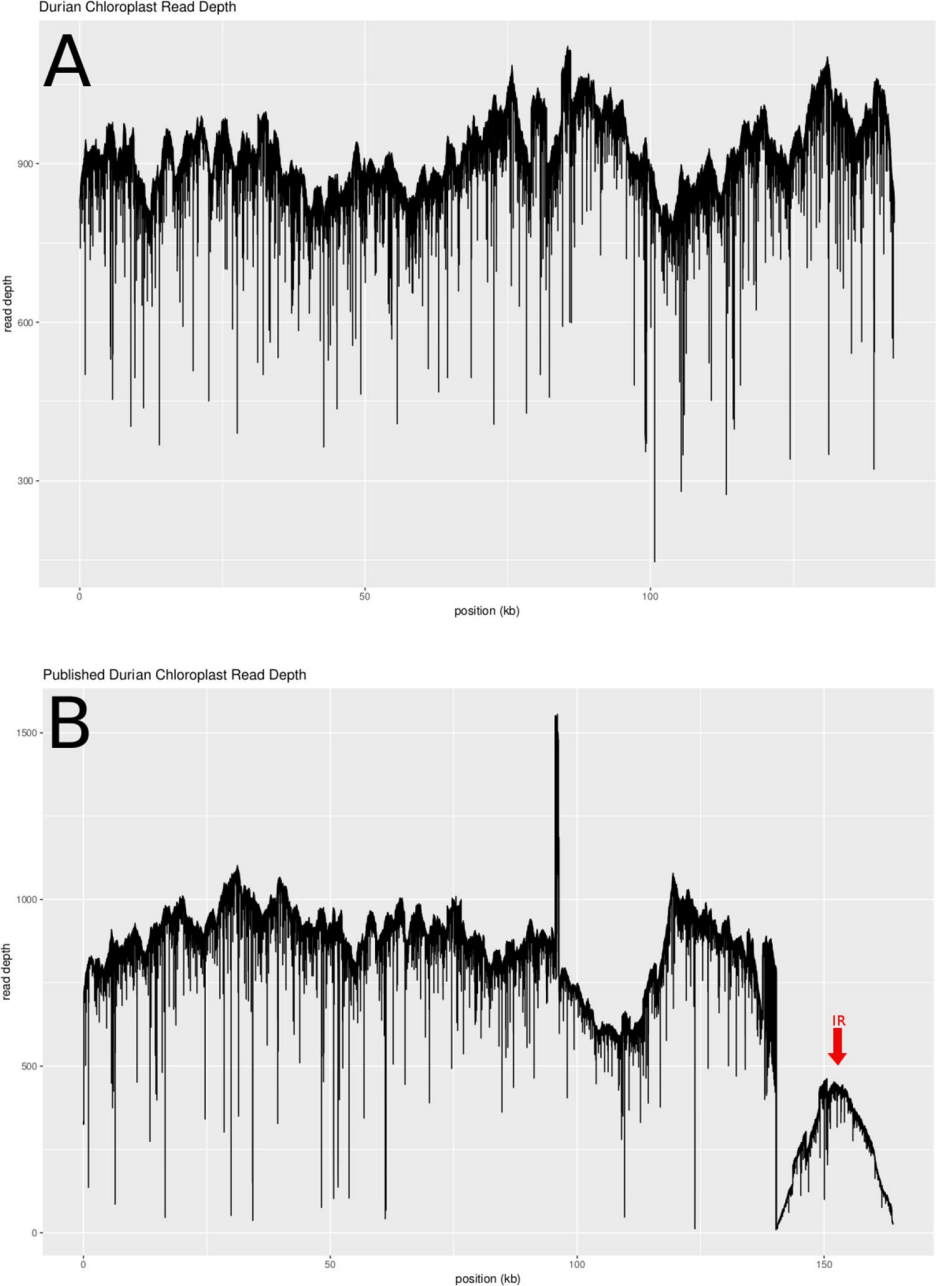
Read depth of long PacBio reads mapped against the published durian chloroplast genome (MG138151.1).

To investigate the absence of the IR, we mapped the long reads (> 10 kb) to both the chloroplast sequence that we generated and to the published chloroplast sequence. The mapped reads showed that our chloroplast assembly was well supported, the long reads mapped with a fairly uniform distribution and greater than 10 kb overlaps between reads for any point along the assembly (supplementary table S1). Multiple long reads confirmed that the contig was cyclic with half of each read mapping to the start of the contig and the other half mapping to the end of the contig (supplementary table S2). In contrast, the published chloroplast assembly had a junction not supported by our sequence data. This unsupported junction is evident from a gradual decline in read depth leading into and out from the junction (Fig. 2). The unsupported junction occurs at the point of the IR that Cheon et al.^2^ reported, which is consistent with the lack of an IR in our assembly. The chloroplast genome tends to have relatively low diversity within a species^4,14,15^, so we do not expect that both assemblies would be correct, especially since they are from the same variety. Interestingly, when we blasted our chloroplast assembly against the published Musang King whole genome assembly^1^, only two chloroplast contigs, totalling 40 kb, were found, meaning that the whole genome assembly lacks the chloroplast genome. This is likely the result of a filtering step during HiC scaffolding.

### Comparison to other durian varieties

**Figure 3:**
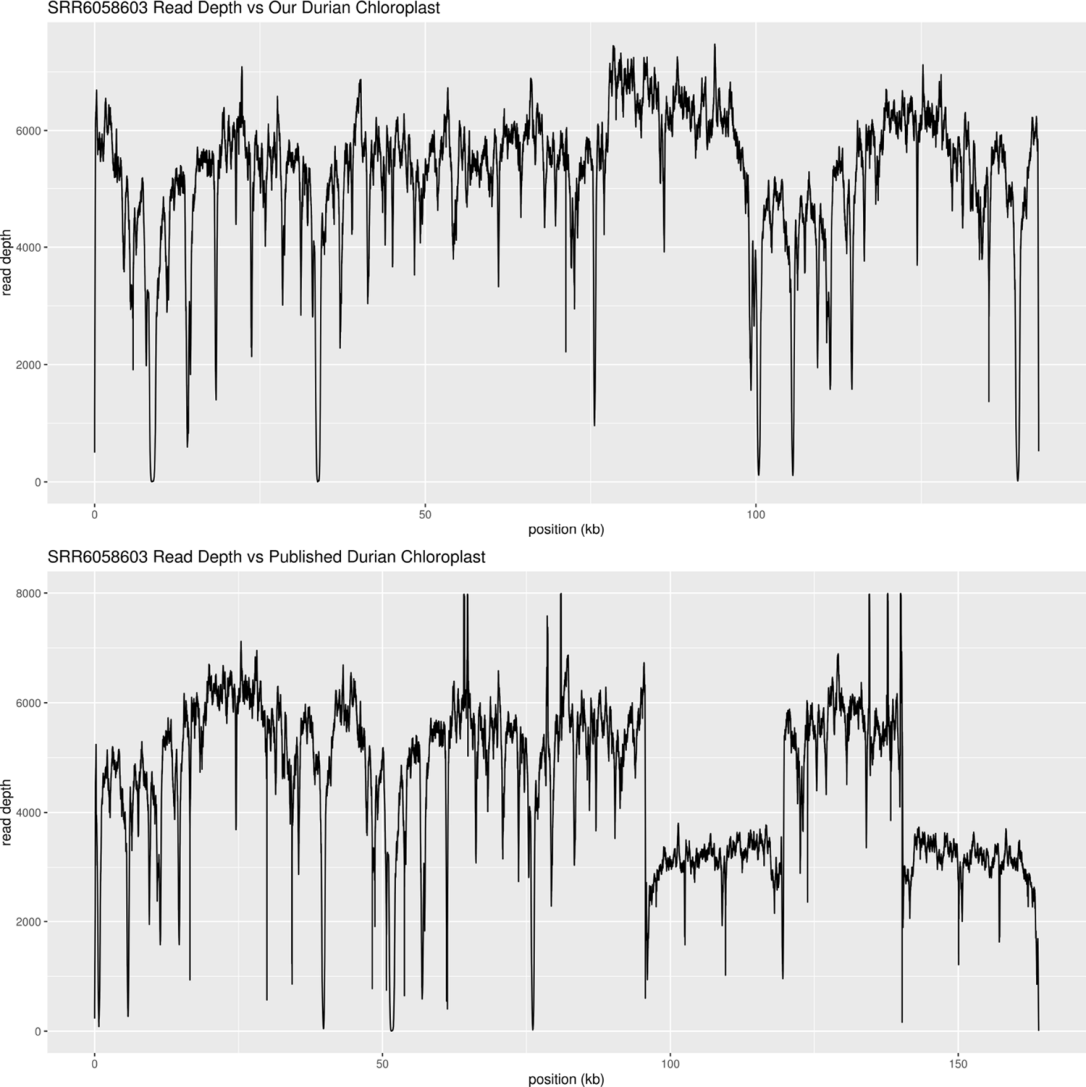
Read depth of Musang King (SRX3204603) against our chloroplast and the published chloroplast genome sequences (MG138151.1).

We performed short read sequencing of 24 Thai varieties (supplementary table S3) of durian and downloaded the Illumina reads from the Musang King variety^1^ (SRX3204603). We mapped these 25 durian varieties to both the published chloroplast assembly and our chloroplast assembly. Repeat regions and regions of extreme GC content (0–20% GC) showed significant drops in read depth in both assemblies (Fig. 3, Supplementary Figure S1-S24). Monthong was also included in the short read sequences and showed the same read depth drops as the other samples, showing that the read depth drops are from the short read sequences and not because of differences in sequence between the samples. The most striking drop in read depth occurred at the IR of the published assembly, with all samples showing approximately half read depth at both copies of the IR (Fig. 3, Supplementary Figure S1-S24). This shows that the IR does not exist in any of the 25 varieties as the reads that should all map to the single copy are being divided between the two, consistent with our assembly showing no IR. However, read depth calling programs tend to set a maximum limit for each position and with high copy number genomes, such as the chloroplast, this number can easily be reached by even a moderate amount of sequence data, resulting in saturation of read depth at each location. Such was the case when we used the full data for the Musang King variety (Supplementary Figure S25), all positions showed fairly equal coverage when default settings were used, and the halved read depth signal was only visible when a small portion of the reads were used.

We called variants in the 24 Thai varieties and the Musang King variety of durian mapped against our version of the chloroplast genome. Since the data is from the whole genome there are also reads from the mitochondria and nuclear genomes, which could be identified based on relative read depth in most cases. There were nine SNPs identified in six of the varieties that could be reliably identified as chloroplast based on read depth (Table 2). We also found 100 variants where the read depth was consistent with chloroplast, yet split between two alleles, suggesting heteroplasmy (supplementary table S4). There are a number of reports of chloroplast heteroplasmy in a variety of species, which is proposed to originate from bi-parental inheritance or through spontaneous mutation^16–19^. These studies mostly used PCR on purified chloroplast fractions, so confounding results from mitochondria or nuclear DNA have been accounted for. Hoang et al.^20^ found that true chloroplast reads accounted for approximately 70% of whole genome shotgun reads that mapped to a chloroplast reference with the other reads coming from nuclear or mitochondria genomes, fairly consistent with what we found, and suggested this could be used to identify cases of heteroplasmy. However, since we did not map to the whole genome, it is possible that these variants occur in high copy number sequence from the mitochondrial and/or nuclear genomes. In addition, there was no overlap with the nine SNPs that could be assigned as chloroplast only. Thus, these variants represent potential heteroplasmic variants, but lack sufficient evidence to confirm true heteroplasmy.

**Table 2:** Chloroplast SNPs identified from 24 Thai varieties and the Musang King variety of duran.

POS	REF	ALT	X41	X43	X46	X62	X79	Mk
4449	C	T	Ref (243,5)	Alt (29,216)	Alt (31,216)	Alt (39,210)	Ref (243,5)	Alt (82,168)
33,109	G	C,T	Ref (242,6,0)	Alt2 (1,24,221)	Alt2 (3,10,236)	Alt2 (8,10,229)	Ref (245,1,0)	Alt2 (60,0,190)
33,936	G	A,T	Ref (36,0,0)	Alt2 (0,11,76)	Alt2 (0,18,108)	Alt2 (1,8,54)	Ref (89,2,5)	Alt2 (19,0,25)
36,163	A	T	Alt (33,216)	Ref (245,5)	Ref (247,1)	Ref (249,1)	Ref (247,3)	Ref (245,1)
36,164	A	T	Alt (5,244)	Ref (239,11)	Ref (245,5)	Ref (248,2)	Ref (241,7)	Ref (246,0)
36,165	A	T	Alt (14,233)	Ref (240,9)	Ref (244,5)	Ref (246,3)	Ref (243,7)	Ref (248,1)
37,110	G	T	Ref (236,5)	Alt (8,201)	Alt (0,213)	Alt (1,207)	Ref (232,7)	Alt (44,151)
37,111	A	C,G,T	Ref (214,0,16,7)	Alt (22,201,1,25)	Alt (19,213,1,16)	Alt (16,213,0,18)	Ref (231,0,9,6)	Ref (144,87,0,19)
134,508	T	A	Ref (248,1)	Alt (38,205)	Alt (27,211)	Alt (31,211)	Ref (248,2)	Alt (77,171)

Call per sample is indicated as Ref: same as our chloroplast assembly; or Alt, Alt2; first or second allele in the ALT column, respectively. Number of reads that support each allele are given in the brackets in the order Ref, Alt, Alt2, Alt3.

### Comparison with other species

**Figure 4:**
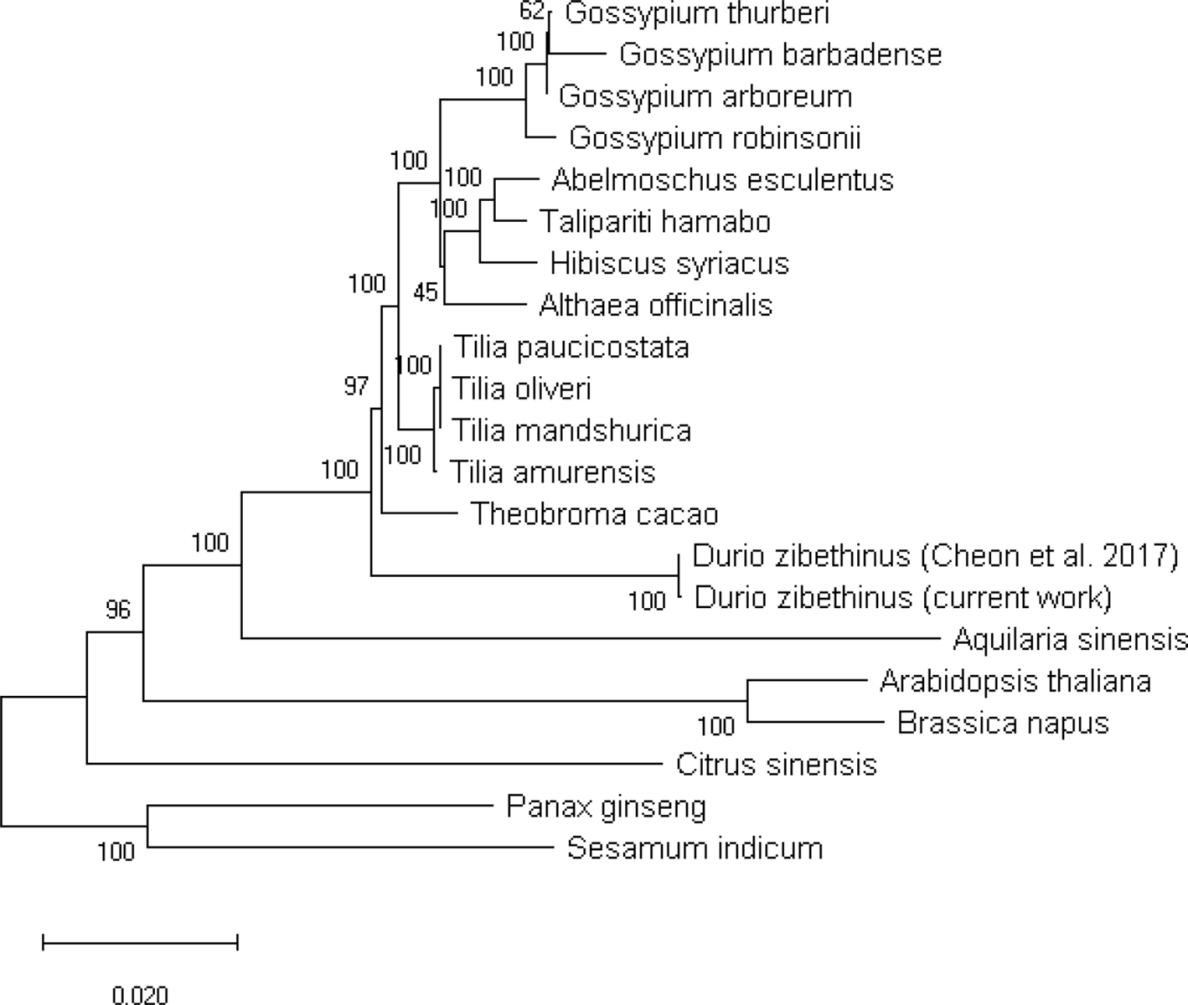
Phylogenetic tree using chloroplast genes.

The protein sequences of the durian chloroplast genes were compared to 19 other species plus the published durian chloroplast and a phylogenetic tree was constructed (Fig. 4). The results show durian sharing ancestry with *Tilia* species and *Theobroma cacao*, consistent with analyses performed using the whole genome assembly^1^ and the chloroplast genome^2^. All of the closest species to durian are reported to contain an IR, so the loss of the IR must have occurred after durian split from these species. It should be noted, however, that the chloroplast genomes for these species were also assembled from short read data, which, considering our findings, raises some doubt regarding their degree of accuracy.

## Conclusions

We have assembled the durian chloroplast from the Monthong variety using long PacBio reads that spanned all low complexity sequence allowing for the whole chloroplast genome to be assembled into a single high-confidence contig. We found, despite a publication showing an IR being present, that the durian chloroplast lacks the IR that is common to plant chloroplast genomes. We then used publicly available short read data to show that it can be difficult to identify the assembly error using only short read data, which highlights the value of using long read data for de novo assembly.

## Materials and methods

### Sample and DNA extraction

The durian sample is a Monthong variety maintained at the Chantaburi Horticultural Research Center, Thailand. Leaf tissue was collected from a single plant and used for DNA extraction with the standard CTAB method. The DNA sample was subsequently purified with Ampure PB beads (Pacific Biosciences, Menlo Park, USA), and the DNA integrity was assessed using the Pippin Pulse Electrophoresis System (Sage Science, Beverly, USA).

### Sequencing and assembly

DNA was used to prepare libraries for the PacBio RSII following the Pacific Biosciences ‘Procedure and Checklist-20 kb Template Preparation Using BluePippin Size-Selection System’ protocol. DNA (10 ug) was sheared with a Covaris gTube, 4500 rpm for 2 min and the BluePippin cassette used was ‘0.75%DF Marker S1 high-pass 15–20 kb’ with selection of 12–50 kb. Sequencing was performed for 14 cells on the PacBio RSII. Raw reads longer than 20 kb were used as seed reads and reads shorter than 20 kb were used to correct them by the RS_PreAssembler.1 protocol with default settings from the Pacific Biosciences SMRTanalysis (v2.3.0) software package. The corrected reads were then assembled using CANU (version 1.8)^12^ and ABruijn assembler (version 2.0b)^13^. Quiver (part of the SMRTanalysis suite) was then run on the final assembly to fix PacBio sequencing errors. Annotation was performed using the online tool CpGAVAS^21^. The genome was plotted using OGDraw^2﻿2^. Repeats were identified by blasting the assembly against itself.

The published chloroplast genome assembly^2^ and the raw reads from the whole genome assembly of the Musang King variety durian^1^ (SRX3204603) were downloaded from NCBI. All of our corrected reads and the SRX3204603 reads were then mapped to our assembly and the published assembly, using BWA MEM for the PacBio reads and bowtie^23^ for the Illumina reads, to confirm that the assembly was supported by the majority of reads. Read depth was calculated from each set of mapping data using samtools depth and plotted using ggplot2 in R.

### Sequence comparison and phylogenetic tree

A phylogenetic tree was constructed using 19 species (*Gossypium thurberi*, *Gossypium barbadense*, *Gossypium arboreum*, *Gossypium robinsonii*, *Abelmoschus esculentus*, *Talipariti hamabo*, *Hibiscus syriacus*, *Althaea officinalis*, *Tilia paucicostata*, *Tilia oliveri*, *Tilia mandshurica*, *Tilia amurensis*, *Theobroma cacao*, *Aquilaria sinensis*, *Arabidopsis thaliana*, *Brassica napus*, *Citrus sinensis*, *Panax ginseng*, *Sesamum indicum*). Gene sequences from each species for 111 common genes (Table 3) were compared and a phylogenetic tree was constructed using MEGA-X with the maximum likelihood method and bootstrap 1000 times^24^.

**Table 3:** List of genes that were used to construct a phylogenetic tree.

Genes used to construct phylogenetic tree				
rpl21	rpoC2	trnR-ACG	psbI	petL
rpl14	rrn4.5	trnR-UCU	psbJ	petN
rpl16	rrn5	trnS-GCU	psbK	atpA
rpl20	rrn16	trnS-UGA	psbL	atpB
rpl232	rrn23	trnS-GGA	psbM	atpE
rpl32	trnA-UGC	trnT-UGU	psbN	atpF1
rpl33	trnC-GCA	trnT-GGU	psbT	atpH
rpl36	trnD-GUC	trnV-GAC	psbZ	atpI
rps2	trnE-UUC	trnV-UAC	ndhA	rbcL
rps3	trnF-GAA	trnW-CCA	ndhB1	matK
rps4	trnfM-CAU	trnY-GUA	ndhC	cemA
rps7	trnG-GCC	psaA	ndhD	accD
rps8	trnG-UCC	psaB	ndhE	ccsA
rps11	trnH-GUG	psaC	ndhF	clpP1
rps121	trnI-CAU	psaI	ndhG	ycf1
rps14	trnI-GAU	psaJ	ndhH	ycf2
rps15	trnL-CAA	psbA	ndhI	ycf31
rps161	trnL-UAG	psbB	ndhJ	ycf4
rps18	trnL-UAA	psbC	ndhK	ycf15
rps19	trnM-CAU	psbD	petA	
rpoA	trnN-GUU	psbE	petB	
rpoB	trnP-UGG	psbF	petD	
rpoC11	trnQ-UUG	psbH	petG	

## Supplementary information

Supplementary file1

## Data Availability

The durian chloroplast assembly is available at Genbank accession: MT321069. The raw sequence data is available at Genbank BioProject ID: PRJNA625389.
