# Supplementary material for: Assembly of the durian chloroplast genome using long PacBio reads
Source: Sci Rep. 2020 Oct 7;10:15980. doi: 10.1038/s41598-020-73549-4 (PMC7541610; doi:10.1038/s41598-020-73549-4)

Assembly of the durian chloroplast genome using long PacBio reads

Jeremy R. Shearman[1], Chutima Sonthirod[1], Chaiwat Naktang[1], Duangjai Sangsrakru[1], Thippawan Yoocha[1], Ratchanee Chatbanyong[2], Siriporn Vorakuldumrongchai[2], Orwintinee Chusri[2], Sithichoke Tangphatsornruang[1], Wirulda Pootakham[1,*]

[1] National Omics Center, National Center for Genetic Engineering and Biotechnology, National Science and Technology Development

Agency, 113 Thailand Science Park, Paholyothin Road, Khlong Nueng, Khlong Luang, Pathumthani, 12120, Thailand

[2] Chantaburi Horticultural Research Center, Department of Agriculture, Chanthaburi, 22110, Thailand

[*] Corresponding author

Supplementary Tables and Figures

Supplementary Table S1: Subset of the longest PacBio reads showing full coverage and large read overlap of the durian chloroplast genome assembly

ReadId	match	identity	align.len	mismatches	gaps	q.start	q.end	s.start	s.end	E value	Bit score	Read Length
10202	Durian_chloroplast	99.81	51147	2	89	1	51076	2197	53317	0	91477	51076
18069	Durian_chloroplast	99.51	34331	14	128	1	34203	6782	41087	0	60720	34206
509	Durian_chloroplast	99.79	27070	3	37	1	27029	12706	39762	0	48443	27029
6301	Durian_chloroplast	99.61	28675	9	65	1	28610	13657	42294	0	51011	28610
9750	Durian_chloroplast	99.83	26038	2	35	1	26000	16035	42068	0	46638	26000
24053	Durian_chloroplast	99.58	30154	6	93	1	30043	17054	47196	0	53492	30043
17662	Durian_chloroplast	99.74	34100	18	62	2	34052	18389	52465	0	60875	34052
17165	Durian_chloroplast	99.53	26601	10	73	1	26512	20114	46688	0	47182	26512
21065	Durian_chloroplast	99.77	28115	3	53	1	28064	21513	49615	0	50223	28064
17915	Durian_chloroplast	99.87	26998	0	29	1	26968	24864	51857	0	48434	26968
1872	Durian_chloroplast	99.86	25154	1	29	1	25129	27430	52574	0	45105	25129
6494	Durian_chloroplast	99.85	27557	1	32	1	27524	27549	1	0	49399	28730
2653	Durian_chloroplast	99.95	25118	3	9	1	25109	27713	52830	0	45211	25109
7381	Durian_chloroplast	99.95	25492	0	14	1	25479	28224	53714	0	45859	25479
15242	Durian_chloroplast	99.78	30145	2	55	1	30089	30150	60287	0	53878	30089
14860	Durian_chloroplast	99.68	27152	16	57	3	27104	32459	5330	0	48376	27104
22006	Durian_chloroplast	99.84	25236	0	32	1	25201	32972	7743	0	45218	25201
2086	Durian_chloroplast	99.77	32485	4	52	1	32425	34695	67169	0	58078	32425

2169	Durian_chloroplast	99.63	27120	3	73	1	27028	34791	61906	0	48219	27028
9087	Durian_chloroplast	99.83	28584	0	37	1	28539	35257	6678	0	51204	28539
18789	Durian_chloroplast	99.88	32762	2	33	1	32727	35355	68114	0	58791	32727
16314	Durian_chloroplast	99.83	27465	0	42	1	27423	36387	63845	0	49168	27423
963	Durian_chloroplast	99.21	30226	14	185	1	30007	38343	8123	0	52803	30007
12310	Durian_chloroplast	99.47	28821	4	126	1	28682	39091	10282	0	50849	28682
9009	Durian_chloroplast	99.74	29502	4	65	2	29446	41157	70641	0	52625	29446
7205	Durian_chloroplast	99.88	27429	2	29	1	27402	41375	68799	0	49213	27402
23824	Durian_chloroplast	99.87	29516	0	34	1	29488	44311	14805	0	52942	29488
2182	Durian_chloroplast	99.77	25622	4	36	1	25575	44944	19330	0	45832	25575
20648	Durian_chloroplast	99.88	25827	0	27	1	25799	45384	19560	0	46346	25799
10787	Durian_chloroplast	99.75	29791	1	64	1	29724	45548	75332	0	53168	29724
1097	Durian_chloroplast	99.86	27662	1	34	1	27630	46411	74066	0	49590	27630
17383	Durian_chloroplast	99.85	35008	6	39	1	34965	46857	81861	0	62763	34965
24195	Durian_chloroplast	99.41	29658	12	127	1	29506	47253	17607	0	52270	29506
20621	Durian_chloroplast	99.65	28495	17	67	1	28442	50033	78496	0	50706	28442
600	Durian_chloroplast	99.74	27028	2	39	1	26972	50164	23148	0	48315	26972
11343	Durian_chloroplast	99.89	25326	4	23	1	25304	50780	25457	0	45464	25304
3418	Durian_chloroplast	99.88	28956	0	27	1	28929	51136	80084	0	51974	28929
18288	Durian_chloroplast	99.86	33881	0	40	1	33838	52238	86112	0	60744	33838
8816	Durian_chloroplast	99.84	26809	1	36	1	26778	53264	80062	0	48032	26778
22992	Durian_chloroplast	99.83	26877	0	39	2	26837	54045	80916	0	48127	26837
16645	Durian_chloroplast	99.82	25505	0	42	4	25468	54441	79940	0	45644	25468
17397	Durian_chloroplast	99.68	25084	10	37	1	25018	54833	29754	0	44771	25018
23939	Durian_chloroplast	99.55	28025	5	103	1	27910	55948	27930	0	49617	27910
17006	Durian_chloroplast	99.64	25275	5	80	1	25194	57095	31827	0	44883	25194
2389	Durian_chloroplast	99.72	25626	5	59	1	25575	58789	84397	0	45680	25575
1558	Durian_chloroplast	99.85	28352	0	37	1	28315	59329	30984	0	50807	28315
15241	Durian_chloroplast	99.73	32186	10	63	1	32129	62341	30177	0	57433	32129
1576	Durian_chloroplast	99.85	30132	1	41	1	30108	62778	92889	0	53992	30108
580	Durian_chloroplast	99.88	26571	0	30	1	26543	63345	36778	0	47671	26543
15399	Durian_chloroplast	99.85	37446	1	32	1	37412	63378	100803	0	67185	37412
2759	Durian_chloroplast	99.83	25385	0	39	1	25344	64334	89717	0	45451	25344
24874	Durian_chloroplast	99.95	27219	0	13	1	27207	64538	91754	0	48977	27207
5213	Durian_chloroplast	99.85	28884	0	34	1	28844	65983	37104	0	51777	28844
9154	Durian_chloroplast	99.89	29016	0	21	1	28986	67153	96165	0	52113	28986
10810	Durian_chloroplast	99.65	25886	14	45	1	25844	68381	94232	0	46142	25844
1373	Durian_chloroplast	99.87	29353	0	31	1	29318	68781	98131	0	52661	29318

8050	Durian_chloroplast	99.9	25472	0	26	1	25446	69122	94593	0	45725	25446
8532	Durian_chloroplast	99.61	37307	7	83	1	37210	71094	108359	0	66376	37210
11172	Durian_chloroplast	99.75	28796	1	37	1	28747	71701	100475	0	51507	28747
4548	Durian_chloroplast	99.45	28289	6	127	1	28162	72084	100350	0	49878	28162
11396	Durian_chloroplast	99.91	25216	3	18	1	25200	73464	98676	0	45310	25200
21519	Durian_chloroplast	99.77	26135	3	39	1	26087	74666	48540	0	46740	26087
3481	Durian_chloroplast	99.83	25662	0	37	1	25633	75127	49481	0	45952	25633
7291	Durian_chloroplast	99.72	35040	4	54	1	34971	75434	110448	0	62590	34971
18872	Durian_chloroplast	99.9	31088	0	30	1	31060	77947	46863	0	55817	31060
23224	Durian_chloroplast	99.84	25482	2	33	1	25444	78371	52892	0	45651	25444
2658	Durian_chloroplast	99.16	30603	4	116	1	30379	80271	110845	0	53739	30379
8236	Durian_chloroplast	99.48	29207	10	85	1	29068	81858	52654	0	51734	29068
8979	Durian_chloroplast	99.89	29559	1	28	1	29530	82116	52561	0	53060	29530
24335	Durian_chloroplast	99.84	33744	3	46	1	33698	82419	48682	0	60444	33698
8695	Durian_chloroplast	99.82	28578	1	42	1	28533	82675	54103	0	51162	28537
2144	Durian_chloroplast	99.64	25030	5	61	1	24989	84283	109269	0	44539	24989
20468	Durian_chloroplast	99.89	29652	0	26	1	29623	84412	54766	0	53234	29623
5581	Durian_chloroplast	99.79	37931	0	51	1	37871	85184	123094	0	67885	37871
10247	Durian_chloroplast	99.77	27343	3	34	3	27320	85543	112850	0	48926	27320
8922	Durian_chloroplast	99.76	28564	1	50	1	28499	89133	60572	0	51040	28502
17055	Durian_chloroplast	99.84	29388	2	41	1	29347	90978	61594	0	52645	29347
16826	Durian_chloroplast	99.9	25218	0	17	1	25201	91099	65889	0	45314	25201
6011	Durian_chloroplast	99.82	27169	0	30	1	27142	91782	118927	0	48681	27142
17421	Durian_chloroplast	99.79	28418	5	41	1	28382	94907	123305	0	50843	28382
9465	Durian_chloroplast	99.65	31002	6	80	1	30922	94992	125972	0	55157	30922
18297	Durian_chloroplast	99.8	39102	8	60	1	39039	95563	56471	0	69950	39039
7349	Durian_chloroplast	99.78	28091	0	42	1	28050	95972	124041	0	50246	28050
9155	Durian_chloroplast	99.89	27990	5	22	1	27970	96190	68208	0	50257	27970
9580	Durian_chloroplast	99.88	27425	0	30	1	27396	98713	71292	0	49208	27396
24063	Durian_chloroplast	99.05	29436	38	130	1	29208	101253	71833	0	51451	29208
3548	Durian_chloroplast	99.67	26139	10	47	1	26095	102017	75912	0	46603	26095
23417	Durian_chloroplast	99.6	33332	8	100	1	33226	103211	136522	0	59169	33226
2682	Durian_chloroplast	99.63	26662	18	51	1	26590	104626	131279	0	47482	26590
5334	Durian_chloroplast	99.71	31976	7	64	1	31932	104638	72705	0	57034	31932
6647	Durian_chloroplast	99.63	35396	1	97	1	35291	104646	69275	0	62926	35291
1132	Durian_chloroplast	99.54	25388	5	89	1	25299	106509	131872	0	44953	25300
5492	Durian_chloroplast	99.68	27286	4	34	1	27221	108391	81125	0	48733	27221
1551	Durian_chloroplast	99.88	26847	1	31	1	26823	109503	136341	0	48156	26823

736	Durian_chloroplast	99.7	30284	7	52	1	30230	110195	79942	0	54044	30230
11530	Durian_chloroplast	99.84	30072	7	30	1	30048	112695	142750	0	53920	32039
23729	Durian_chloroplast	99.83	25329	1	37	1	25291	112764	138089	0	45359	25293
11186	Durian_chloroplast	99.84	28379	0	37	1	28338	112790	141165	0	50852	28338
1101	Durian_chloroplast	99.84	25696	0	33	1	25658	113682	139374	0	46043	25658
6792	Durian_chloroplast	99.86	25663	1	29	1	25635	113705	139359	0	46016	25635
17309	Durian_chloroplast	99.72	25824	7	40	1	25766	113800	139616	0	46124	25766
12008	Durian_chloroplast	99.76	27608	1	42	3	27565	114852	87265	0	49359	27565
16240	Durian_chloroplast	99.64	26679	8	67	1	26598	116080	142750	0	47453	26941
13153	Durian_chloroplast	99.9	26283	2	21	1	26261	116469	142750	0	47211	28915
1149	Durian_chloroplast	99.7	27689	2	57	4	27636	118476	90812	0	49379	27636
10413	Durian_chloroplast	99.8	35960	3	49	1	35914	119969	84033	0	64367	35914
21355	Durian_chloroplast	99.85	26107	3	35	1	26075	128379	102277	0	46780	26078
22909	Durian_chloroplast	99.79	25608	0	39	1	25558	128693	103090	0	45810	25558
1461	Durian_chloroplast	99.73	41727	7	72	1	41653	129358	87665	0	74507	41653
23096	Durian_chloroplast	99.82	27044	2	43	1	27001	130254	103216	0	48394	27001
1454	Durian_chloroplast	99.73	30281	0	61	1	30203	133023	102748	0	54033	30203
2464	Durian_chloroplast	99.51	37254	12	125	1	37115	133150	95928	0	65952	37115
17200	Durian_chloroplast	99.69	27232	7	69	3	27167	135317	108097	0	48486	27167
13304	Durian_chloroplast	99.85	26526	0	35	1	26487	135380	108855	0	47538	26487
11635	Durian_chloroplast	99.87	29003	1	30	1	28979	135406	106416	0	52033	28979
1919	Durian_chloroplast	99.89	28597	0	29	1	28575	135612	107025	0	51328	28575
8048	Durian_chloroplast	99.92	31837	0	19	1	31817	142320	110488	0	57242	31817
8064	Durian_chloroplast	99.8	36030	0	62	3252	39221	142750	106733	0	64437	39221
21264	Durian_chloroplast	99.96	27147	0	11	1586	28721	142750	115605	0	48863	28721

Supplementary Table S2: Subset of the longest PacBio reads showing the cyclic nature of the durian chloroplast genome assembly

Read Id	match	identity	align.len	mismatches	gaps	q.start	q.end	s.start	s.end	E value	Bit score	Read Length
1119	Durian_chloroplast	99.82	25833	2	35	1	25799	25823	1	0	46261	29787
1119	Durian_chloroplast	99.38	4008	0	22	25800	29787	142750	138748	0	7039	29787
1844	Durian_chloroplast	99.83	25904	1	38	1	25870	25894	1	0	46380	30194
1844	Durian_chloroplast	99.33	4343	6	20	25871	30194	142750	138412	0	7633	30194
8064	Durian_chloroplast	99.57	3264	0	14	1	3251	3263	1	0	5773	39221
8064	Durian_chloroplast	99.8	36030	0	62	3252	39221	142750	106733	0	64437	39221
11726	Durian_chloroplast	99.73	7117	0	19	1	7100	7115	1	0	12680	34293
11726	Durian_chloroplast	99.64	27279	5	55	7101	34291	142750	115476	0	48593	34293

13694	Durian_chloroplast	99.66	8172	0	19	1	8147	8169	1	0	14550	27962
13694	Durian_chloroplast	99.86	19838	0	20	8148	27962	142750	122917	0	35588	27962
17952	Durian_chloroplast	99.66	15486	3	29	1	15440	15483	1	0	27607	27773
17952	Durian_chloroplast	98.89	12436	19	54	15441	27773	142750	130331	0	21668	27773
20313	Durian_chloroplast	99.87	8554	0	11	1	8545	8552	1	0	15337	28540
20313	Durian_chloroplast	99.89	20017	0	16	8546	28540	142750	122734	0	35947	28540
21112	Durian_chloroplast	99.87	8460	0	11	3	8455	8456	1	0	15167	25690
21112	Durian_chloroplast	99.87	17242	2	11	8456	25690	142750	125523	0	30959	25690

Supplementary Table S3: Thai name, English name and sample number of the 24 Thai durian varieties that were sequenced

**Number**	**Thai name**	**English name**
41	สายหยุด	Suwai Yut
42	ก้านยาว	Ganyao
43	ก้านยาววัดสัก	Ganyao Wat Sak
44	ก้านยาวสีนาค	Ganyao Sinak
45	ชมภูพาน	Chomphu Phan
46	ทองสุก	Thongsuk
47	หมอนทอง	Monthong
48	กำปั่นเดิม (กำปั่นขาว)	Gumpan Deum
57	กำปั่นพวง	Gumpan Phuang
58	ฉัตรสีทอง	Chat Sithong
59	ธรณีไหว	Thoraniwai
60	นกหยิบ	Nokyip
61	นมสวรรค์	Nomsawan
62	ทองย้อยฉัตร	Thong Yoi Chat
63	กระดุมทอง	Gratum Thong
64	พวงมณี	Phuang Mani
73	กบตาขำ	Kop Thakham

74	กบตาท้วม	Kop Thathuam
75	กบแม่เฒ่า	Kop Maethao
76	กบสุวรรณ	Kop Suwan
77	กบวัดกล้วย	Kop Watkluai
78	ชมพูศรี	Chomphu Si
79	ลวง	Luang
80	ย่ำมะหาด	Yum Mahaat

Supplementary Table S4: Potentially heteroplasmic chloroplast SNPs identified from 24 Thai varieties and the Musang King variety of duran. Call per sample is indicated as Ref: same as our chloroplast assembly; or Alt, Alt2; first or second allele in the ALT column, respectively. Samples that appear heteroplasmic are given both allele calls. Number of reads that support each allele are given in the brackets in the order Ref, Alt, Alt2

POS	REF	ALT	X41	X43	X46	X62	X75	X79	Mk
36	A	T	Ref (224,24)	Ref (210,39)	Ref (220,27)	Ref (215,34)	Ref/Alt (142,108)	Ref (171,78)	Ref (249,0)
41	A	C	Ref (213,37)	Ref (181,67)	Ref (201,48)	Ref (192,56)	Ref/Alt (100,149)	Alt (86,162)	Ref (250,0)
54	A	C	Ref (205,43)	Ref (193,55)	Ref (197,52)	Ref (192,58)	Ref/Alt (138,112)	Ref/Alt (138,111)	Ref (248,1)
55	C	A	Ref (186,62)	Ref (175,73)	Ref (186,61)	Ref (173,76)	Ref/Alt (137,111)	Ref/Alt (129,120)	Ref (250,0)
64	G	A	Ref (216,33)	Ref (197,52)	Ref (215,33)	Ref (196,54)	Ref (165,83)	Ref/Alt (146,103)	Ref (250,0)
66	G	C	Ref (185,63)	Ref (184,65)	Ref (194,54)	Ref (179,70)	Ref (166,84)	Ref/Alt (140,108)	Ref (250,0)
73	A	C	Ref (230,15)	Ref (196,53)	Ref (214,35)	Ref (201,49)	Ref/Alt (121,128)	Ref/Alt (146,103)	Ref (247,0)
74	C	A	Ref (227,23)	Ref (187,61)	Ref (209,40)	Ref (204,44)	Ref/Alt (117,131)	Ref/Alt (133,117)	Ref (247,2)
88	C	G	Ref (217,18)	Ref (183,53)	Ref (220,18)	Ref (208,36)	Ref/Alt (129,112)	Ref (180,64)	Ref (249,0)
121	C	A	Ref (215,33)	Ref (190,58)	Ref (192,57)	Ref (203,44)	Ref (166,84)	Ref/Alt (140,108)	Ref (248,0)
123	A	C	Ref (212,37)	Ref (181,68)	Ref (195,54)	Ref/Alt (148,101)	Ref/Alt (152,96)	Ref/Alt (152,98)	Ref (250,0)
269	C	A,G	Ref/Alt (130,93,26)	Ref (194,28,28)	Ref (162,66,22)	Ref (160,48,42)	Ref (190,25,35)	Ref (194,17,39)	Ref (205,43,0)
1736	C	G	Ref (225,24)	Ref (203,46)	Ref (212,38)	Ref (183,65)	Ref (155,94)	Ref/Alt (148,102)	Ref (247,0)
2386	C	G	Ref (231,18)	Ref (201,49)	Ref (209,39)	Ref (208,42)	Ref (162,88)	Ref/Alt (149,100)	Ref (246,2)
13956	G	A	Ref (166,6)	Ref (238,7)	Ref (238,10)	Ref (246,3)	Ref (119,12)	Ref (174,8)	Ref/Alt (113,133)
13960	T	A	Ref (154,4)	Ref (238,9)	Ref (238,11)	Ref (244,4)	Ref (114,2)	Ref (163,5)	Ref/Alt (117,114)
14496	T	A	Ref (248,1)	Ref (160,85)	Ref (154,95)	Ref/Alt (144,104)	Ref (248,1)	Ref (248,1)	Ref (208,18)
14498	T	A	Ref (242,6)	Ref (137,73)	Ref (137,80)	Ref/Alt (129,94)	Ref (248,1)	Ref (248,2)	Ref (213,16)
14500	T	A	Ref (248,1)	Ref/Alt (128,121)	Ref/Alt (134,115)	Ref/Alt (121,128)	Ref (248,1)	Ref (248,2)	Ref (177,71)
14501	T	A	Ref (246,2)	Ref/Alt (120,127)	Ref/Alt (116,131)	Ref/Alt (120,128)	Ref (249,1)	Ref (246,3)	Ref (162,88)
22199	A	G,T	Ref (238,3,9)	Ref/Alt (141,82,27)	Ref/Alt (145,90,11)	Ref/Alt (143,83,22)	Ref (196,14,39)	Ref (198,14,38)	Ref (166,82,0)
22202	A	C,G	Ref (226,24,0)	Ref (139,55,55)	Ref (135,42,69)	Ref/Alt (139,52,58)	Ref (170,77,2)	Ref (160,86,2)	Ref (156,3,90)
22217	C	A,T	Ref (218,4,28)	Ref/Alt (109,113,28)	Ref/Alt (107,112,30)	Ref/Alt (107,104,39)	Ref (162,10,71)	Ref (161,9,80)	Ref/Alt (141,103,6)
23685	A	G	Ref (245,2)	Ref/Alt (99,118)	Ref/Alt (95,117)	Ref/Alt (93,95)	Ref (247,0)	Ref (245,2)	Ref (173,47)
23869	T	A	Ref (218,32)	Ref (188,60)	Ref (206,43)	Ref (192,55)	Ref (202,45)	Ref/Alt (146,100)	Ref (247,0)
31406	A	T	Ref/Alt (147,102)	Ref (218,29)	Ref (202,47)	Ref (190,59)	Ref (210,39)	Ref (218,32)	Ref (223,24)
31607	T	C,G	Ref/Alt2 (122,31,93)	Ref (180,48,22)	Ref (179,29,39)	Ref (157,37,53)	Ref (166,65,19)	Ref (170,65,15)	Ref (218,0,32)
31654	A	C,T	Ref/Alt (140,93,15)	Ref (208,23,17)	Ref (191,42,17)	Ref (189,48,13)	Ref (203,33,14)	Ref (216,16,18)	Ref (229,21,0)
31933	T	G	Ref/Alt (142,102)	Ref (225,21)	Ref (219,28)	Ref (198,49)	Ref (226,22)	Ref (232,15)	Ref (226,23)
32453	G	A,C	Ref/Alt2 (130,13,106)	Ref (196,19,32)	Ref (168,14,68)	Ref (161,19,69)	Ref (191,32,26)	Ref (188,45,16)	Ref (221,1,28)
32460	A	C,T	Ref/Alt (128,96,22)	Ref (193,32,25)	Ref (171,56,22)	Ref (181,51,18)	Ref (197,23,28)	Ref (172,14,64)	Ref (224,25,1)
32461	C	A,T	Ref/Alt2 (127,10,111)	Ref (191,23,36)	Ref (174,9,67)	Ref (170,17,63)	Ref (202,19,28)	Ref (180,45,23)	Ref (225,1,22)
32684	T	G	Ref/Alt (113,129)	Ref (200,39)	Ref (182,61)	Ref (206,40)	Ref (208,35)	Ref (214,26)	Ref (219,30)

33914	T	A	Ref (27,0)	Ref/Alt (36,33)	Ref/Alt (38,58)	NA	Ref (32,0)	Ref (87,0)	Ref (19,3)
33918	T	A	Ref (28,0)	Ref/Alt (35,26)	Ref/Alt (51,47)	NA	Ref (34,1)	Ref (89,3)	Ref (23,3)
37098	C	T	Ref (214,26)	Ref (224,25)	Ref (227,22)	Ref (222,28)	Ref (204,43)	Ref (215,34)	Ref/Alt (139,97)
39650	C	A,G	Ref/Alt2 (130,46,73)	Ref (168,63,17)	Ref (163,52,33)	Ref (148,60,41)	Ref (138,93,18)	Ref (153,84,11)	Ref (227,0,23)
39688	A	C,T	Ref/Alt (140,76,33)	Ref (175,21,52)	Ref (170,37,41)	Ref (147,41,61)	Ref (169,23,58)	Ref (206,12,32)	Ref (228,22,0)
39692	C	A,T	Ref/Alt2 (121,53,76)	Ref (182,50,17)	Ref (163,53,30)	Ref (151,67,0)	Ref (170,60,20)	Ref (182,53,15)	Ref (228,0,22)
39714	C	A,G	Ref/Alt2 (143,26,80)	Ref (199,30,21)	Ref (179,16,55)	Ref (182,28,40)	Ref (171,64,15)	Ref (188,49,10)	Ref (226,0,23)
39822	A	G	Ref/Alt (135,112)	Ref (201,46)	Ref (180,68)	Ref (161,88)	Ref (168,81)	Ref (180,70)	Ref (225,24)
39943	G	C,T	Ref/Alt2 (136,37,75)	Ref (166,67,15)	Ref (178,39,32)	Ref (164,48,38)	Ref (187,41,21)	Ref (178,58,12)	Ref (234,0,16)
39963	A	C,G	Ref/Alt (136,73,41)	Ref (203,19,27)	Ref (205,26,19)	Ref (190,38,22)	Ref (210,16,23)	Ref (202,16,31)	Ref (239,10,1)
39970	C	A,G	Ref/Alt2 (127,30,93)	Ref (194,29,23)	Ref (185,32,31)	Ref (178,41,31)	Ref (185,44,20)	Ref (184,53,11)	Ref (232,1,17)
40063	A	C	Ref/Alt (143,104)	Ref (204,43)	Ref (189,59)	Ref (188,61)	Ref (193,55)	Ref (188,61)	Ref (232,18)
40201	A	G,T	Ref/Alt (143,96,11)	Ref (203,17,30)	Ref (190,45,15)	Ref (181,45,24)	Ref (177,24,48)	Ref (192,14,44)	Ref (229,17,2)
46763	A	G	Ref (223,26)	Ref (185,64)	Ref (211,36)	Ref (192,56)	Ref (152,98)	Ref/Alt (148,100)	Ref (249,1)
50952	T	G	Ref (208,36)	Ref (160,85)	Ref (190,55)	Ref (173,73)	Ref (168,77)	Ref/Alt (149,100)	Ref (248,0)
52032	A	C	Ref (181,67)	Ref (158,89)	Ref/Alt (146,100)	Ref (160,89)	Ref (191,58)	Ref (187,63)	Ref (249,0)
53028	G	A,C	Ref/Alt (147,73,30)	Ref (201,14,33)	Ref (193,38,18)	Ref (161,57,31)	Ref (179,19,52)	Ref (174,15,61)	Ref (227,23,0)
53045	T	A,C	Ref/Alt2 (145,25,79)	Ref (181,50,17)	Ref (190,23,35)	Ref (158,30,62)	Ref (185,45,19)	Ref (173,63,13)	Ref (230,0,19)
53137	C	A	Ref/Alt (135,98)	Ref (210,33)	Ref (194,50)	Ref (190,51)	Ref (182,59)	Ref (181,60)	Ref (231,11)
53145	A	G	Ref (202,43)	Ref (186,60)	Ref (200,46)	Ref (189,58)	Ref (163,84)	Ref/Alt (147,100)	Ref (248,0)
53310	T	G	Ref/Alt (145,105)	Ref (206,41)	Ref (194,55)	Ref (168,79)	Ref (202,44)	Ref (189,57)	Ref (233,17)
53311	A	T	Ref/Alt (136,97)	Ref (197,46)	Ref (189,50)	Ref (164,73)	Ref (201,46)	Ref (187,56)	Ref (233,16)
53325	A	T	Ref/Alt (129,99)	Ref (207,40)	Ref (184,56)	Ref (166,70)	Ref (208,42)	Ref (191,53)	Ref (230,17)
53326	A	T	Ref/Alt (146,103)	Ref (231,18)	Ref (210,39)	Ref (181,67)	Ref (228,18)	Ref (224,25)	Ref (239,11)
63277	T	C	Ref (217,31)	Ref (168,81)	Ref (193,57)	Ref (171,76)	Ref/Alt (147,101)	Ref (156,93)	Ref (249,1)
75839	T	A	Ref (225,24)	Ref (205,42)	Ref (208,40)	Ref (216,34)	Ref (161,85)	Ref/Alt (149,100)	Ref (249,0)
76101	A	G	Ref/Alt (149,100)	Ref (197,49)	Ref (200,50)	Ref (195,53)	Ref (184,65)	Ref (196,52)	Ref (241,9)
78469	A	C,G	Ref/Alt2 (140,39,70)	Ref (184,32,32)	Ref (180,32,36)	Ref (176,31,43)	Ref (180,35,32)	Ref (197,32,19)	Ref (230,0,19)
78616	G	A,C	Ref/Alt (141,80,29)	Ref (216,14,20)	Ref (186,45,15)	Ref (194,37,19)	Ref (200,22,27)	Ref (205,21,21)	Ref (231,18,1)
78804	A	C,G	Ref/Alt2 (148,19,81)	Ref (205,27,17)	Ref (188,24,35)	Ref (185,19,45)	Ref (178,50,20)	Ref (186,47,16)	Ref (234,0,14)
79387	T	C	Ref/Alt (123,125)	Ref (184,61)	Ref (174,72)	Ref (173,74)	Ref (161,88)	Ref (155,94)	Ref (226,22)
79893	T	G	Ref/Alt (130,118)	Ref (197,42)	Ref (187,56)	Ref (191,54)	Ref (180,63)	Ref (199,44)	Ref (228,22)
81053	T	C	Ref/Alt (132,118)	Ref (189,58)	Ref (185,64)	Ref (163,85)	Ref/Alt (148,101)	Ref (179,71)	Ref (228,19)
84943	T	A,G	Ref/Alt2 (120,42,88)	Ref (178,48,18)	Ref (163,43,38)	Ref (154,44,48)	Ref (172,57,19)	Ref (162,71,15)	Ref (226,0,24)
84944	C	A,T	Ref/Alt (135,83,16)	Ref (208,12,24)	Ref (182,37,25)	Ref (169,42,34)	Ref (178,22,46)	Ref (183,16,47)	Ref (228,22,0)
85695	A	T	Ref (220,29)	Ref (187,62)	Ref (207,43)	Ref (203,47)	Ref/Alt (159,90)	Ref/Alt (148,101)	Ref (249,0)
85846	A	C,T	Ref/Alt2 (138,8,103)	Ref (210,20,20)	Ref (183,23,42)	Ref (219,31,0)	Ref (162,71,17)	Ref (186,46,17)	Ref (232,0,17)
87198	T	C	Ref (195,54)	Ref (151,94)	Ref (168,77)	Ref (159,88)	Ref/Alt (147,102)	Ref (148,98)	Ref (249,0)

98674	G	A	Ref/Alt (128,114)	Ref (201,48)	Ref (160,86)	Ref/Alt (143,106)	Ref (185,63)	Ref (195,50)	Ref (225,25)
99130	A	T	Ref (226,20)	Ref (218,30)	Ref (229,21)	Ref (227,21)	Ref (236,11)	Ref (233,11)	Ref/Alt (139,108)
99134	A	T	Ref (224,25)	Ref (201,49)	Ref (228,21)	Ref (231,17)	Ref (230,18)	Ref (228,15)	Ref/Alt (130,119)
99276	A	T	Ref (233,17)	Ref (226,23)	Ref (232,18)	Ref (241,8)	Ref (233,16)	Ref (235,15)	Ref/Alt (148,99)
101885	G	A,C	Ref/Alt (146,80,24)	Ref (191,26,32)	Ref (185,34,30)	Ref (189,35,26)	Ref (181,24,45)	Ref (178,11,61)	Ref (225,23,0)
105329	G	A	Ref (230,4)	Ref (242,4)	Ref (248,2)	Ref (241,7)	Ref (241,2)	Ref (225,7)	Ref/Alt (136,105)
105334	G	A	Ref (229,1)	Ref (247,2)	Ref (244,4)	Ref (243,6)	Ref (242,2)	Ref (231,3)	Ref/Alt (138,99)
105368	C	A	Ref (211,3)	Ref (238,7)	Ref (243,6)	Ref (243,5)	Ref (222,3)	Ref (218,3)	Ref/Alt (136,104)
109574	A	C	Ref (218,20)	Ref (156,86)	Ref (153,85)	Ref/Alt (148,99)	Ref (191,47)	Ref (205,37)	Ref (249,1)
113885	T	G	Ref (224,22)	Ref (182,59)	Ref (207,39)	Ref (211,34)	Ref/Alt (145,102)	Ref/Alt (148,100)	Ref (246,1)
116800	T	G	Ref (229,19)	Ref (197,50)	Ref (216,32)	Ref (215,34)	Ref (151,97)	Ref/Alt (145,101)	Ref (250,0)
116801	A	T	Ref (222,23)	Ref (189,61)	Ref (209,40)	Ref (207,43)	Ref (150,100)	Ref/Alt (148,101)	Ref (250,0)
129772	C	T	Ref (220,25)	Ref (205,44)	Ref (210,39)	Ref (208,41)	Ref (200,50)	Ref/Alt (147,101)	Ref (250,0)
132975	T	C	Ref (206,43)	Ref (164,85)	Ref (161,86)	Ref/Alt (147,101)	Ref (198,51)	Ref (187,62)	Ref (249,1)
132976	A	T	Ref (209,38)	Ref (159,87)	Ref (165,84)	Ref/Alt (149,101)	Ref (196,51)	Ref (179,61)	Ref (250,0)
133590	A	C	Ref (234,13)	Ref (209,38)	Ref (224,23)	Ref (213,36)	Ref (196,51)	Ref/Alt (147,101)	Ref (249,1)
133591	T	A	Ref (230,20)	Ref (193,57)	Ref (221,28)	Ref (210,39)	Ref (187,62)	Ref/Alt (149,100)	Ref (249,0)
134864	C	A,T	Ref/Alt2 (140,20,88)	Ref (209,15,24)	Ref (190,23,37)	Ref (178,25,47)	Ref (199,18,31)	Ref (215,17,17)	Ref (225,0,23)
134976	A	G	Ref/Alt (118,128)	Ref (204,43)	Ref (193,51)	Ref (181,67)	Ref (197,49)	Ref (213,33)	Ref (219,29)
135012	T	G	Ref/Alt (139,107)	Ref (217,29)	Ref (211,35)	Ref (196,51)	Ref (224,22)	Ref (231,14)	Ref (221,29)
135013	C	A	Ref/Alt (133,102)	Ref (210,33)	Ref (200,35)	Ref (191,51)	Ref (217,22)	Ref (232,13)	Ref (223,26)
135018	T	G	Ref/Alt (144,104)	Ref (217,31)	Ref (206,43)	Ref (196,53)	Ref (227,23)	Ref (231,19)	Ref (218,28)
135056	G	T	Ref/Alt (124,123)	Ref (210,40)	Ref (186,64)	Ref (185,64)	Ref (208,42)	Ref (225,25)	Ref (194,34)
135257	T	A	Ref (145,33)	Ref (167,65)	Ref (184,44)	Ref (172,56)	Ref (186,50)	Ref/Alt (137,105)	Ref (230,6)
135258	C	T	Ref (140,25)	Ref (155,74)	Ref (182,38)	Ref (165,60)	Ref (186,44)	Ref/Alt (136,97)	Ref (227,1)
135453	T	A	Ref (218,31)	Ref (203,45)	Ref (217,32)	Ref (202,46)	Ref (150,96)	Ref/Alt (147,101)	Ref (247,1)
137093	C	G	Ref (219,27)	Ref (209,38)	Ref (226,21)	Ref (227,22)	Ref/Alt (147,102)	Ref (166,80)	Ref (247,0)
139136	T	A	Ref (234,15)	Ref (223,26)	Ref (232,16)	Ref (230,19)	Ref (239,11)	Ref (234,12)	Ref/Alt (137,109)
142016	A	G	Ref (211,37)	Ref (187,61)	Ref (209,35)	Ref (198,48)	Ref (172,77)	Ref/Alt (148,100)	Ref (249,0)

Supplementary Figure S1: Read depth of durian sample 41 Suwai Yut against our chlorplast and the published chloroplast

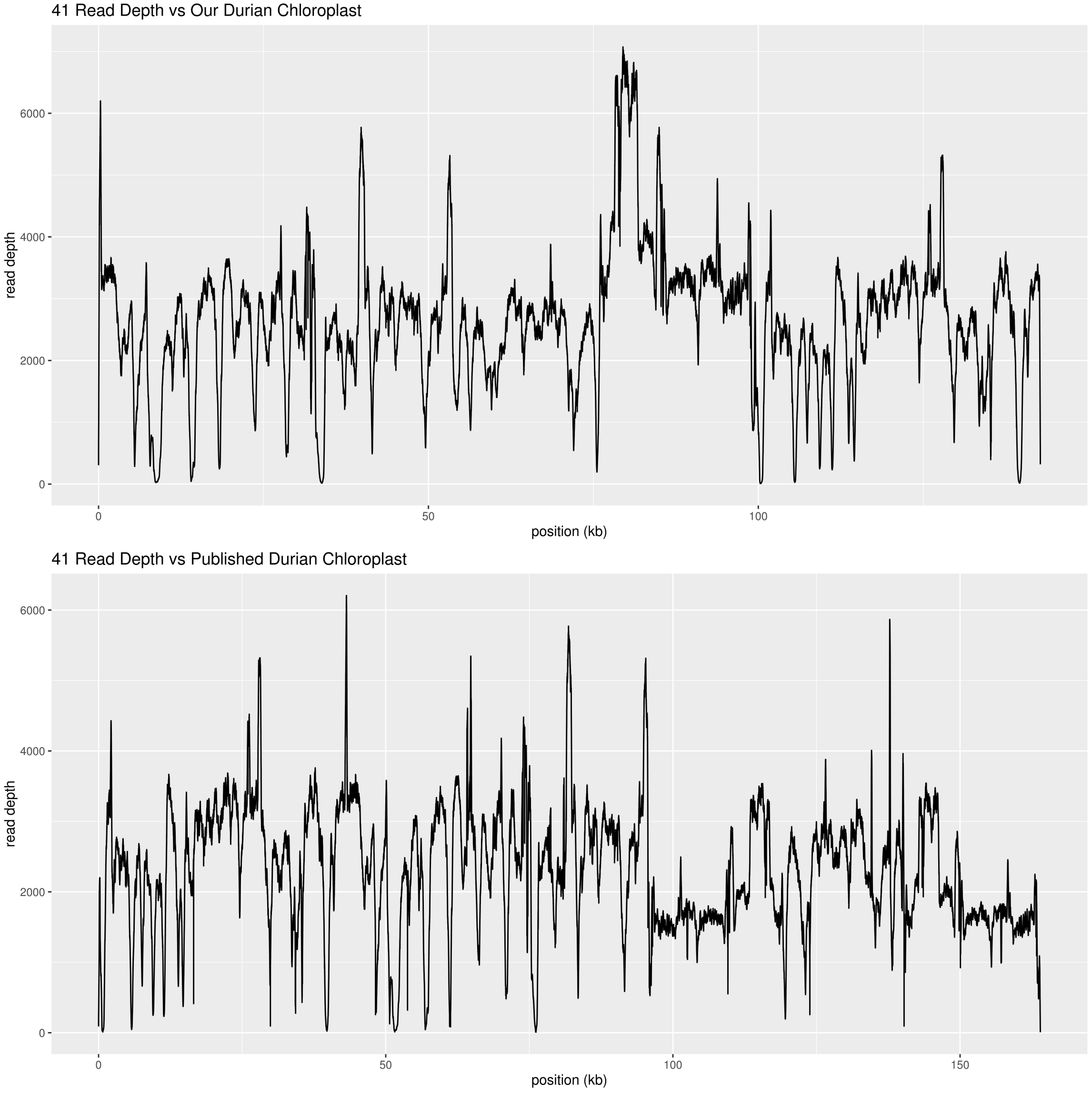

Supplementary Figure S2: Read depth of durian sample 42 Ganyao against our chlorplast and the published chloroplast

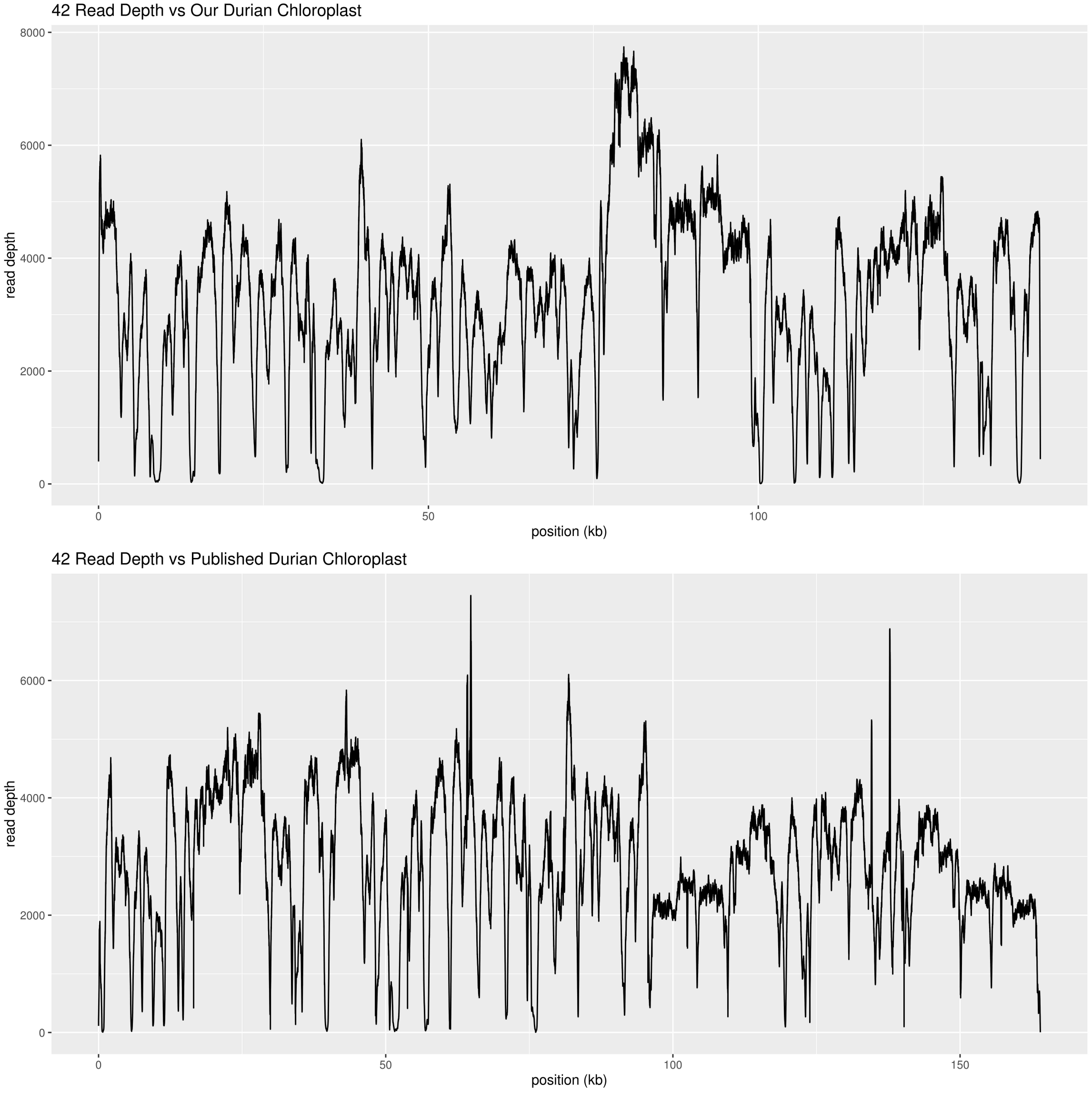

Supplementary Figure S3: Read depth of durian sample 43 Ganyao Wat Sak against our chlorplast and the published chloroplast

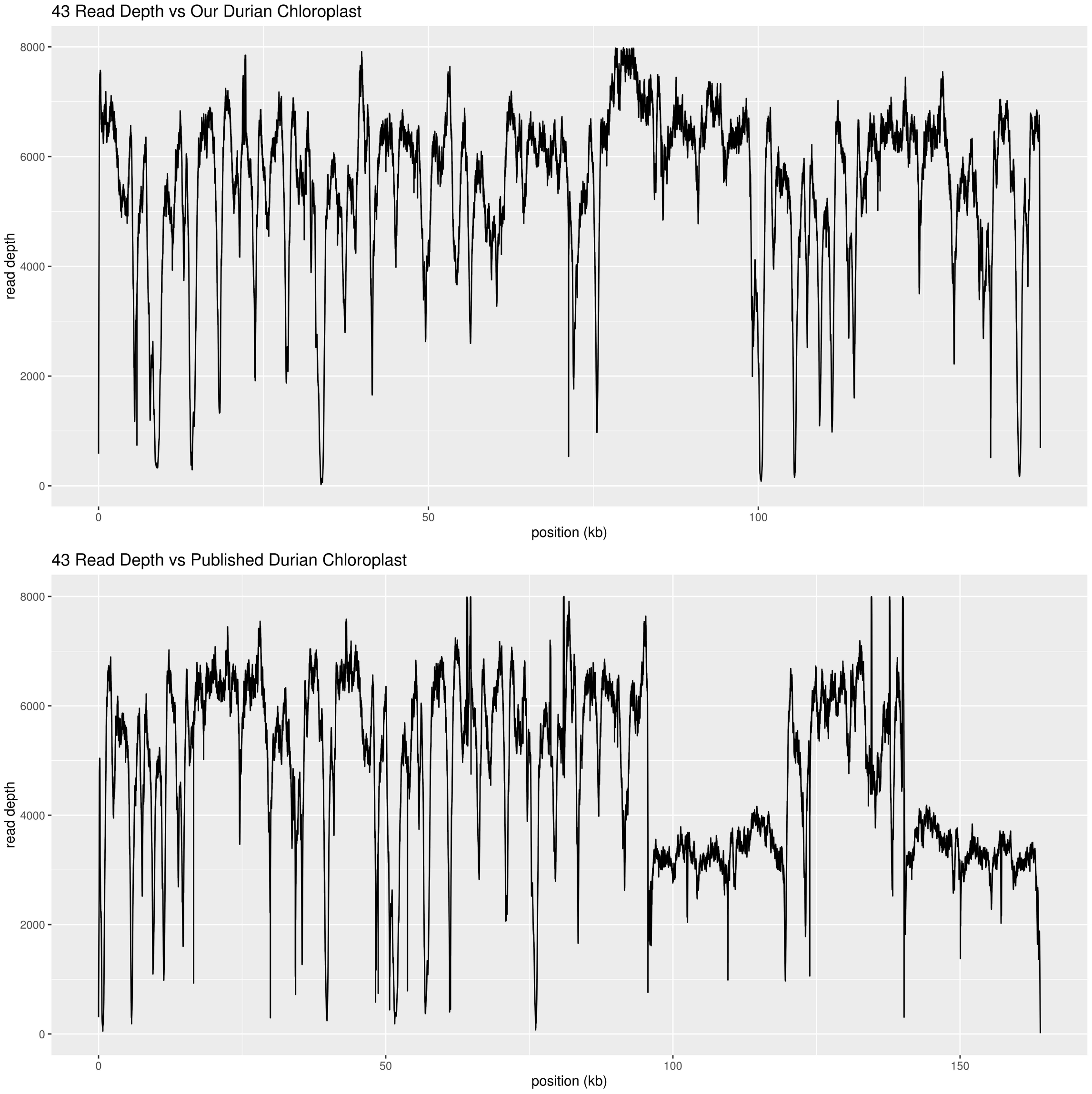

Supplementary Figure S4: Read depth of durian sample 44 Ganyao Sinak against our chlorplast and the published chloroplast

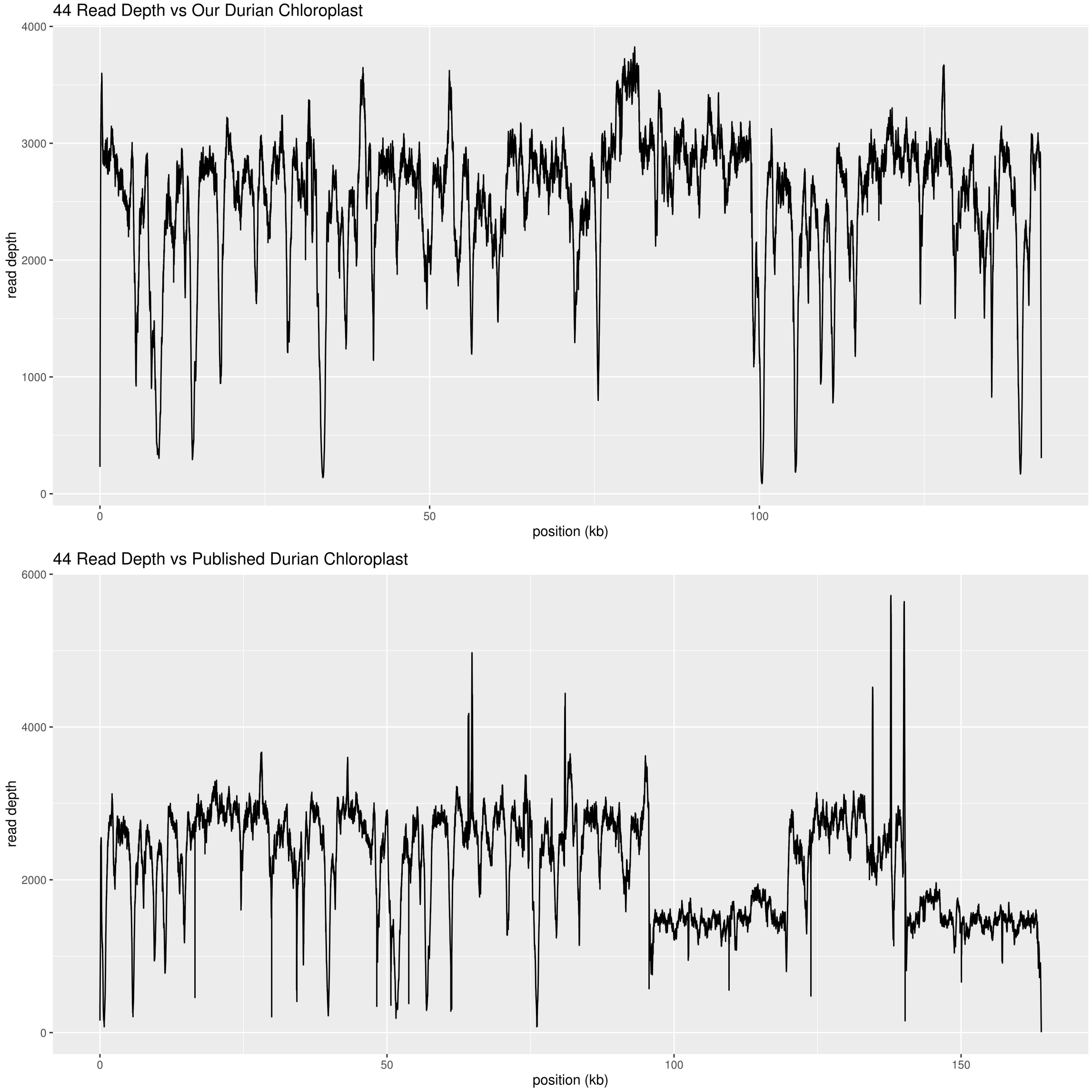

Supplementary Figure S5: Read depth of durian sample 45 Chompu Phan against our chlorplast and the published chloroplast

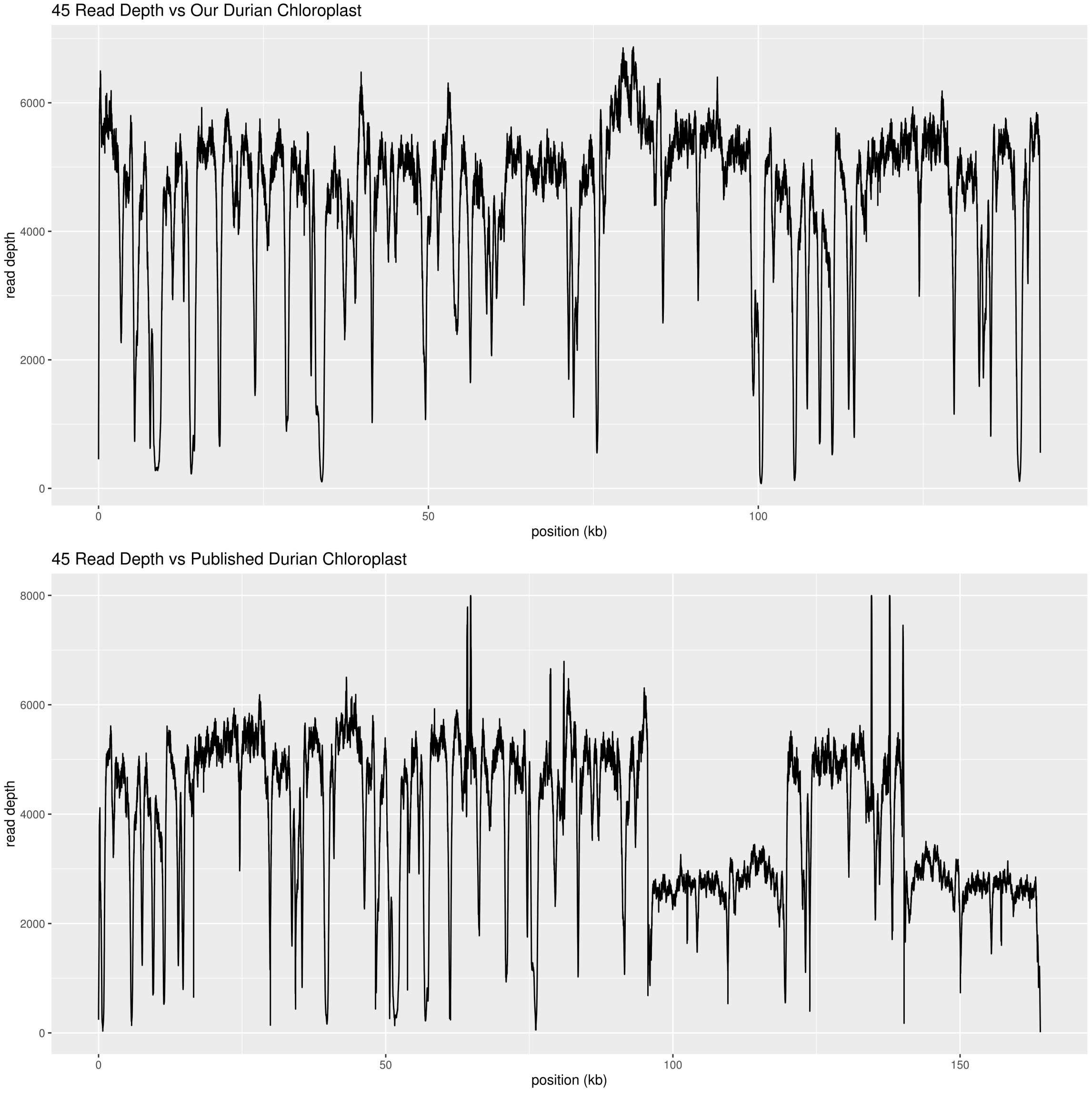

Supplementary Figure S6: Read depth of durian sample 46 Thongsuk against our chlorplast and the published chloroplast

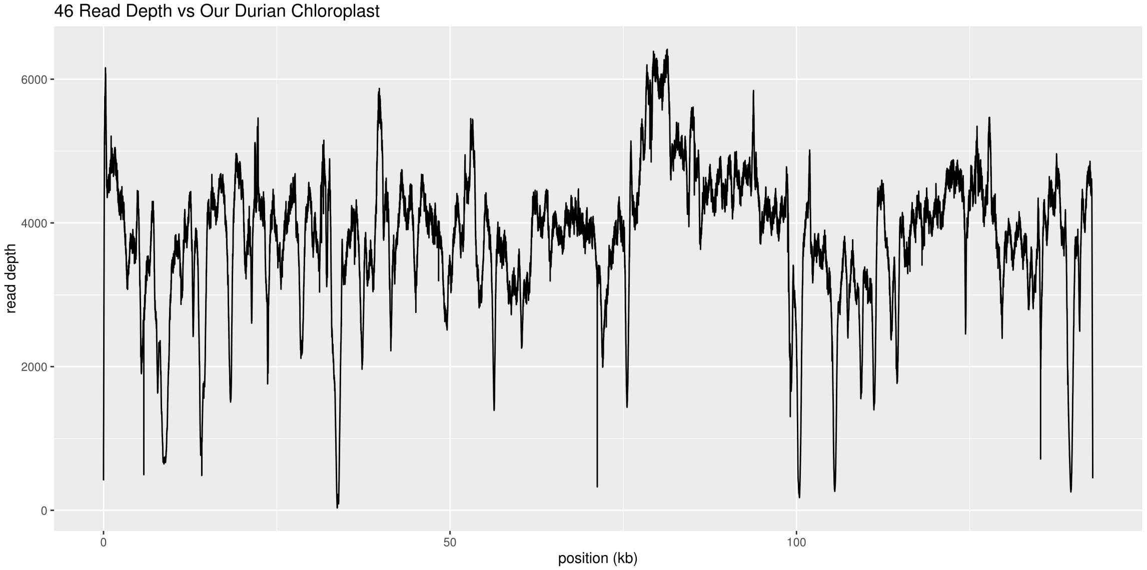

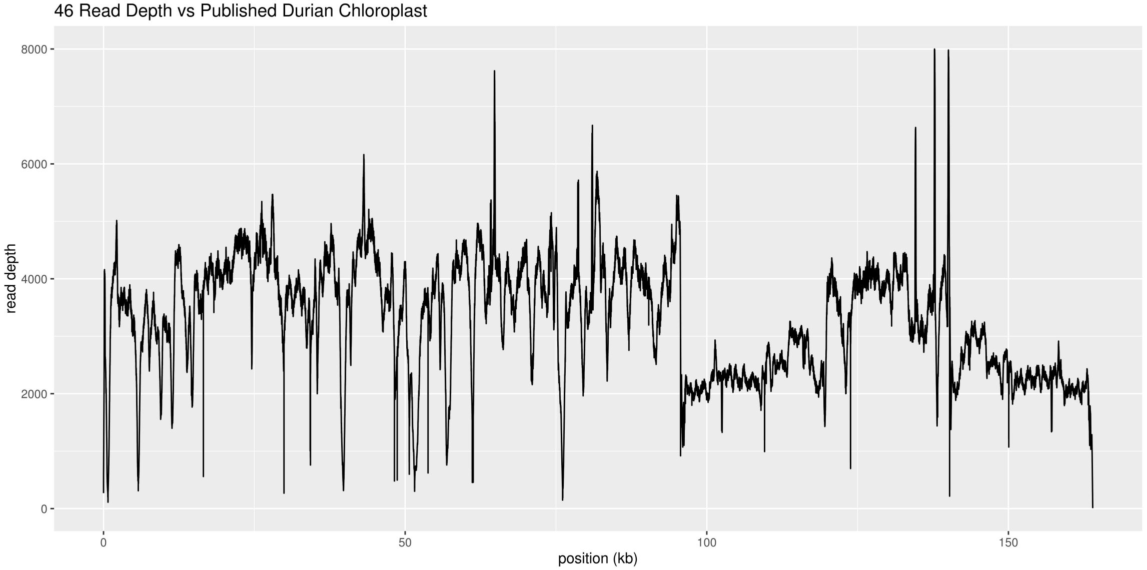

Supplementary Figure S7: Read depth of durian sample 47 Monthong against our chlorplast and the published chloroplast

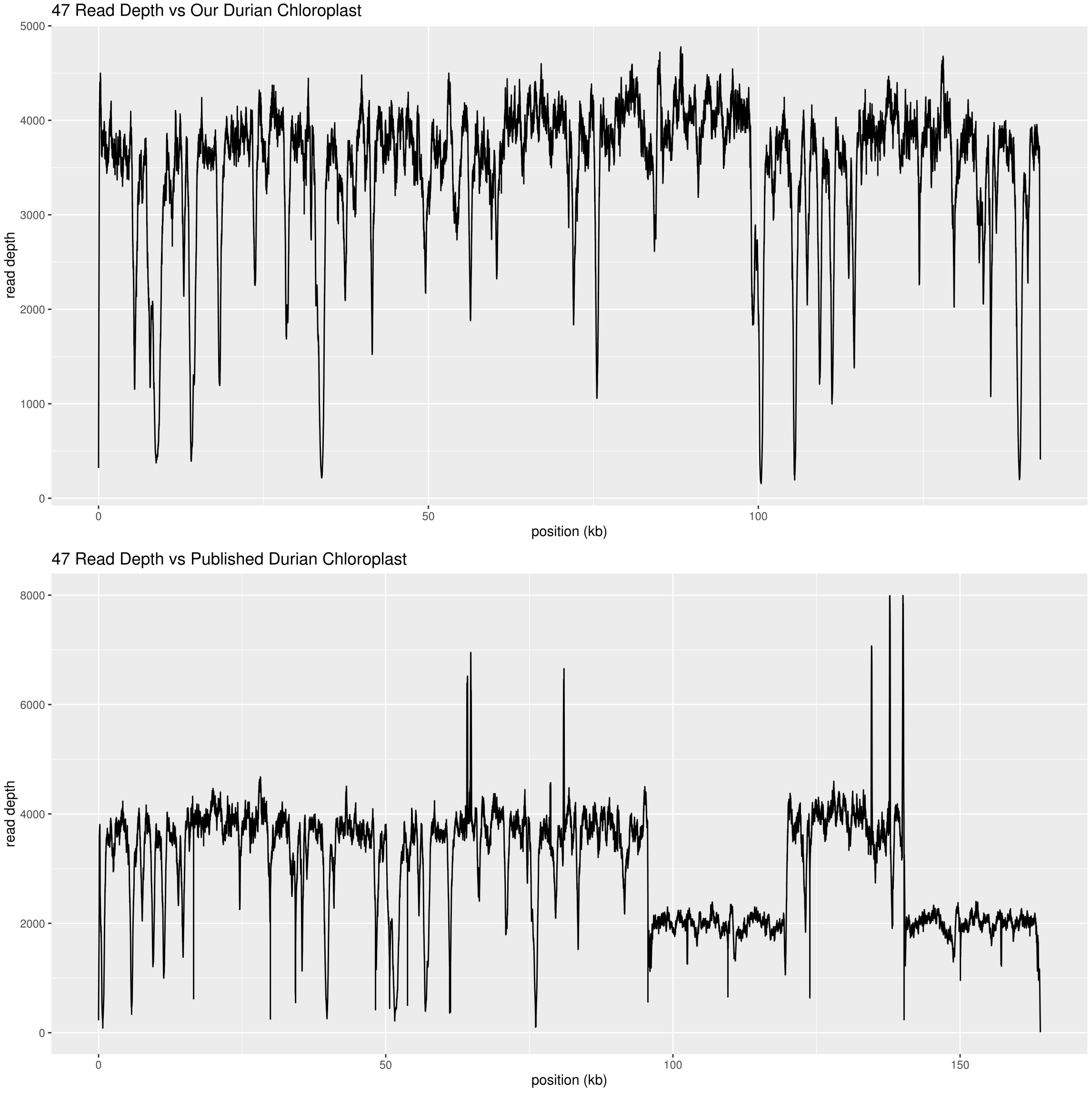

Supplementary Figure S8: Read depth of durian sample 48 Gumpan Deum against our chlorplast and the published chloroplast

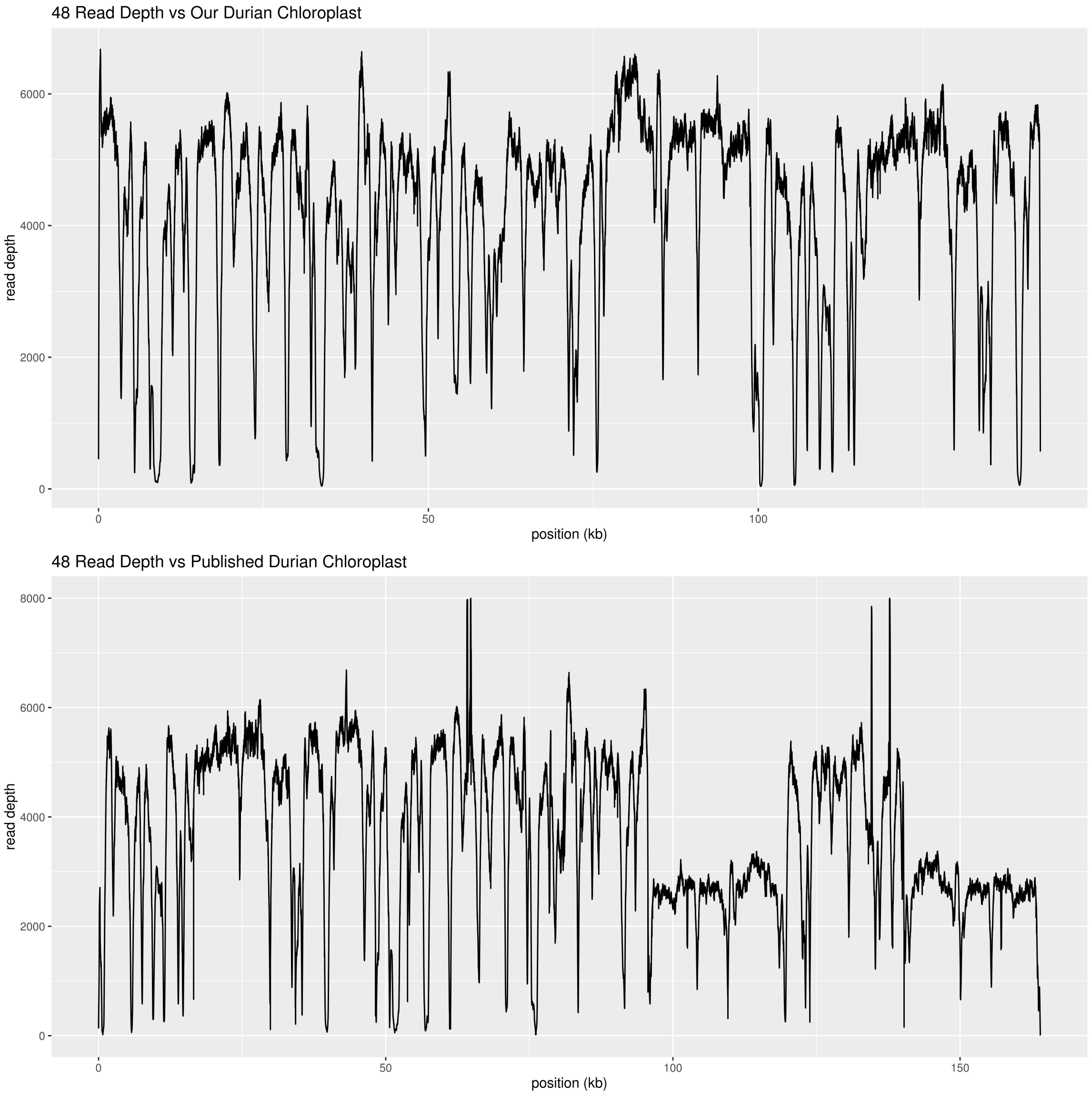

Supplementary Figure S9: Read depth of durian sample 57 Gumpan Phung against our chlorplast and the published chloroplast

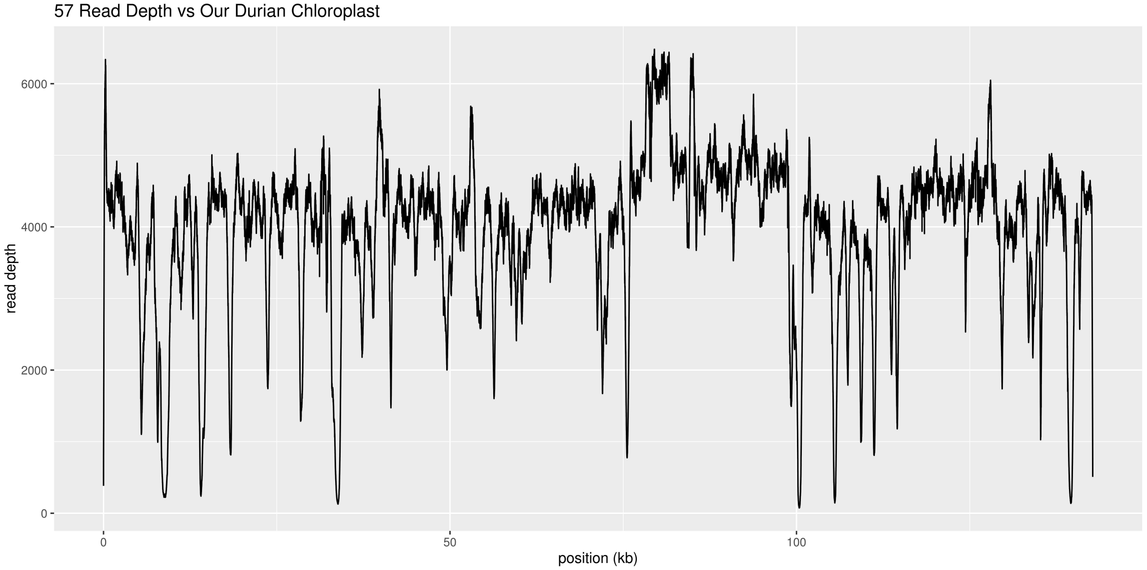

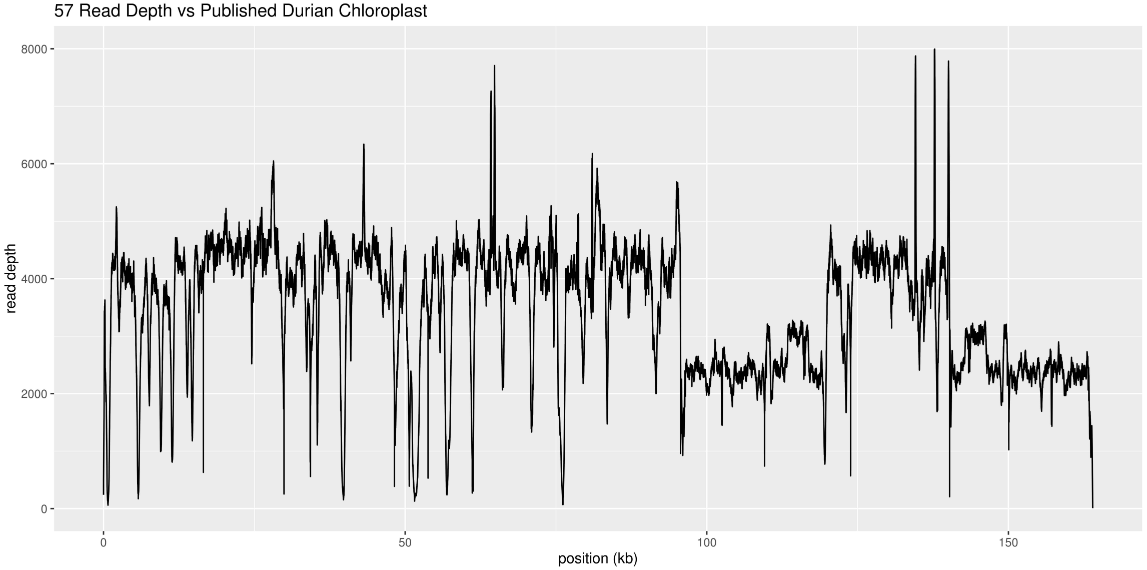

Supplementary Figure S10: Read depth of durian sample 58 Chat Sithong against our chlorplast and the published chloroplast

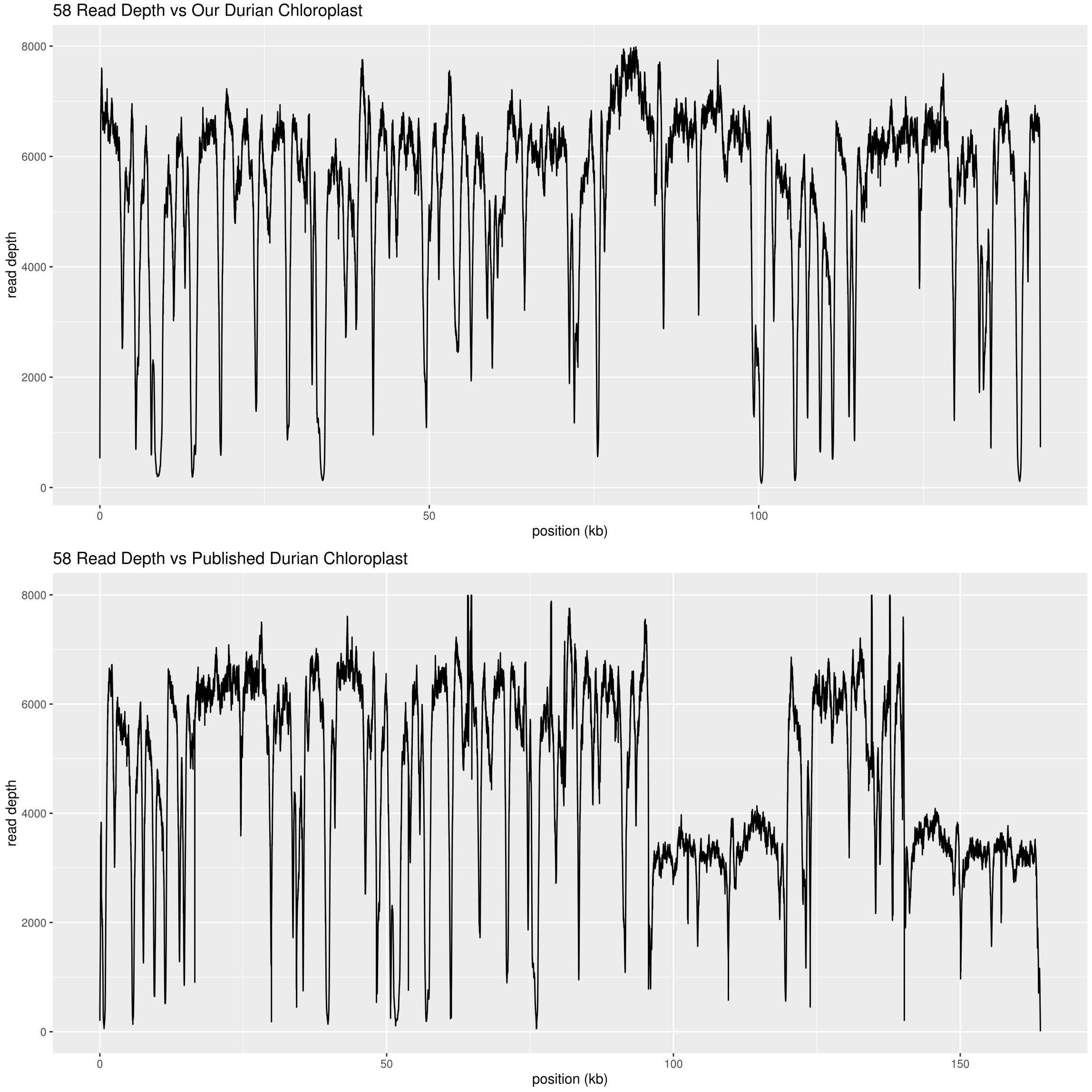

Supplementary Figure S11: Read depth of durian sample 59 Thoraniwai against our chlorplast and the published chloroplast

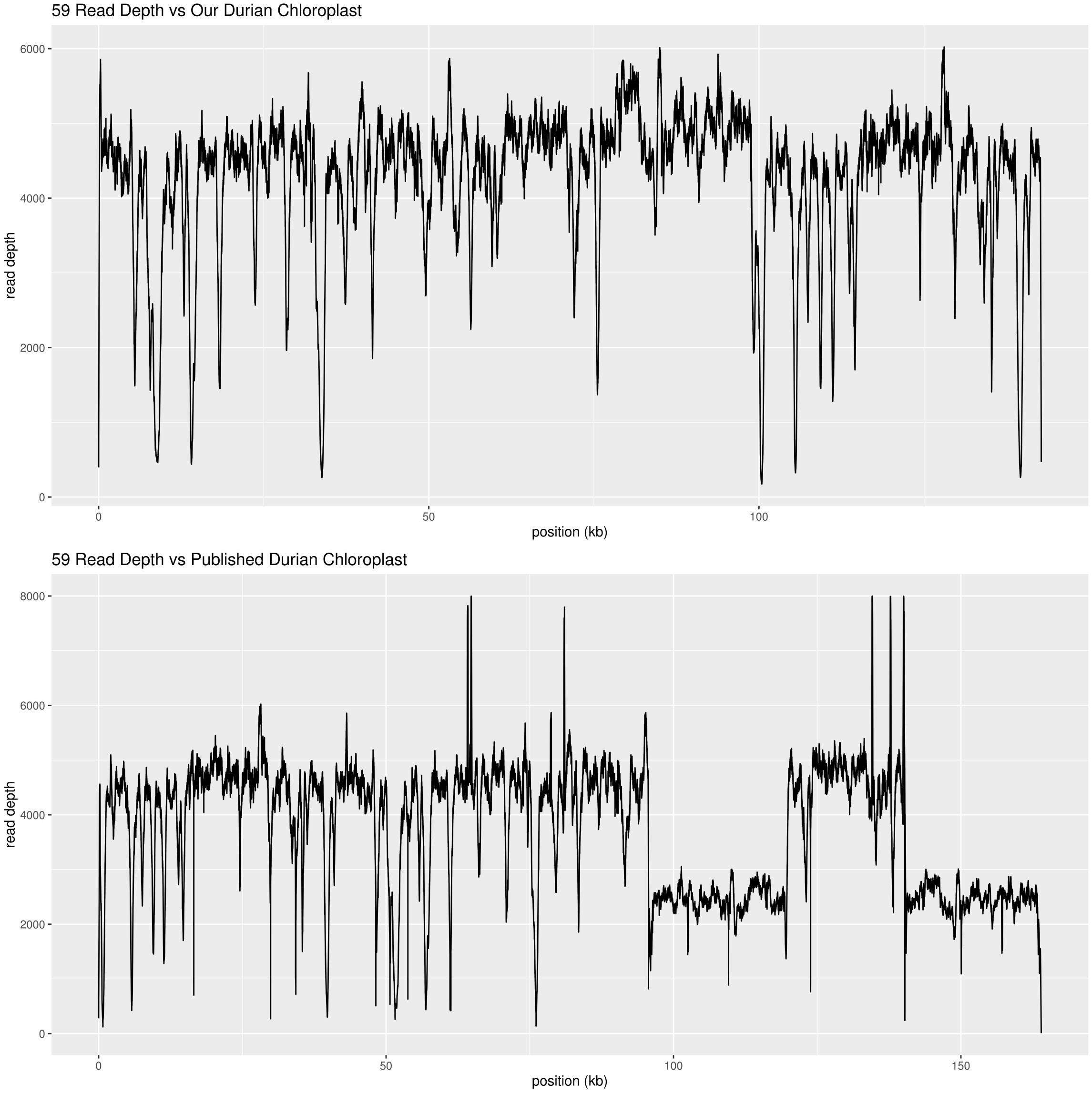

Supplementary Figure S12: Read depth of durian sample 60 Nokyip against our chlorplast and the published chloroplast

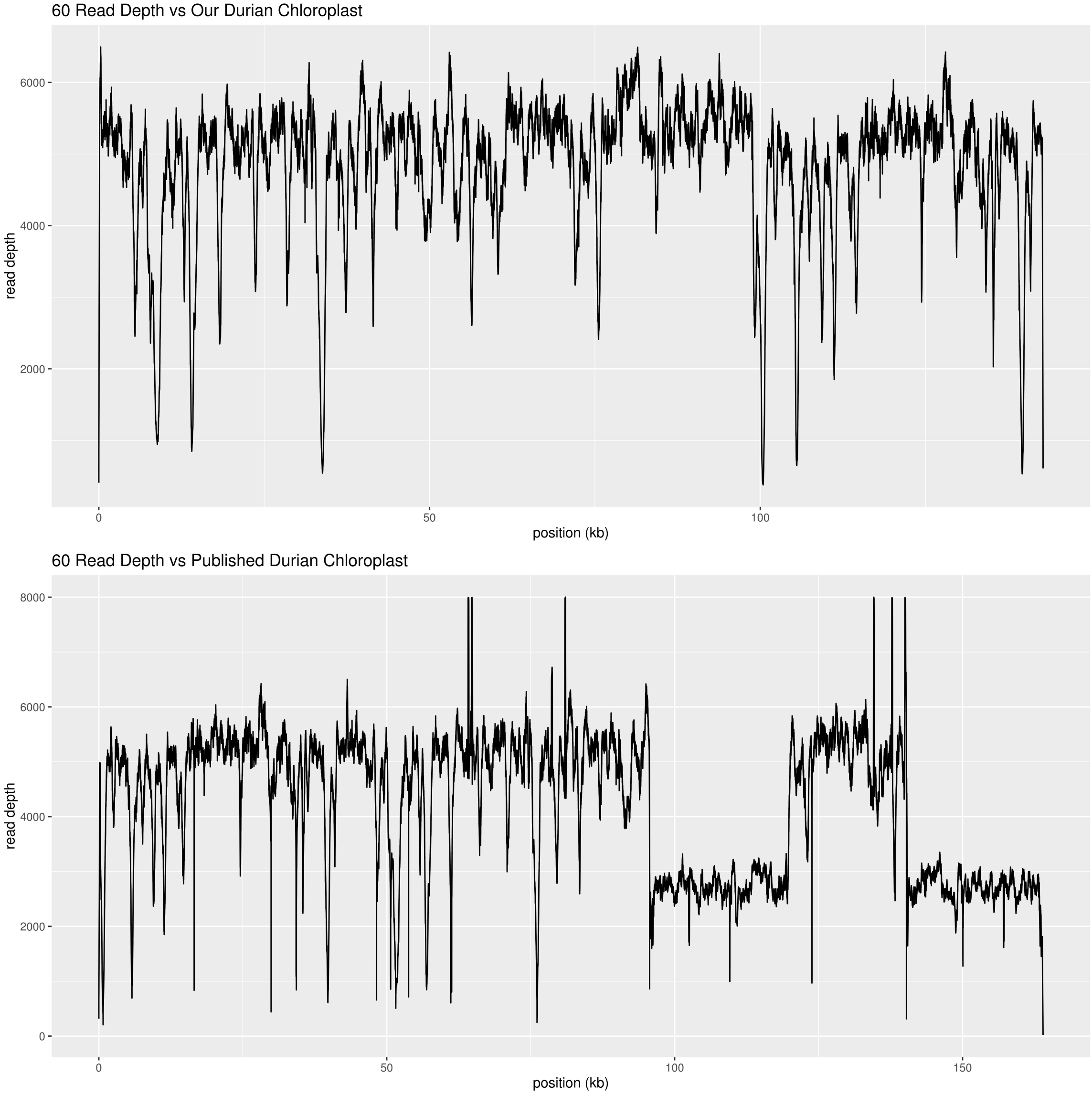

Supplementary Figure S13: Read depth of durian sample 61 Nomsawan against our chlorplast and the published chloroplast

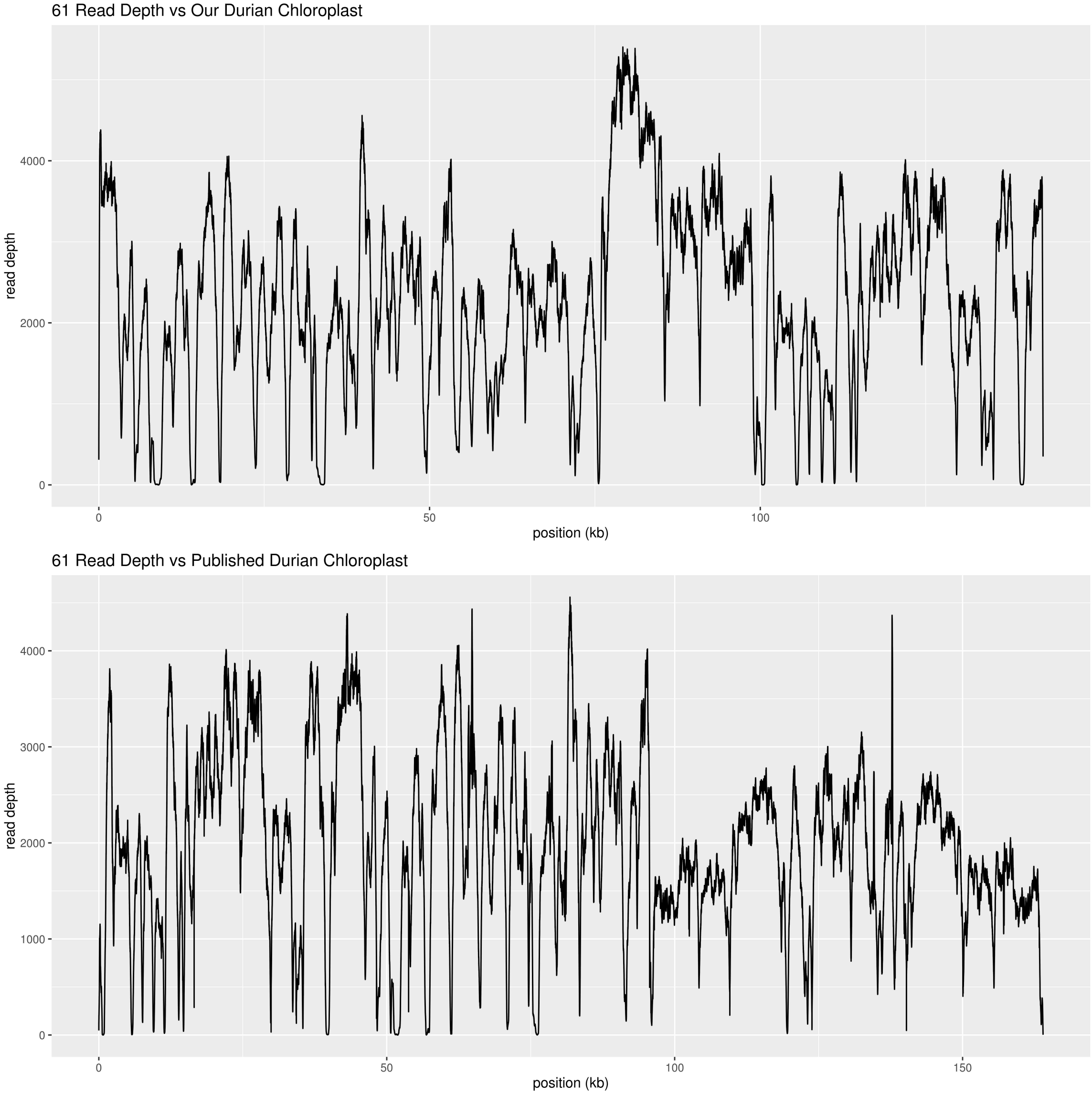

Supplementary Figure S14: Read depth of durian sample 62 Thong Yoi Chat against our chlorplast and the published chloroplast

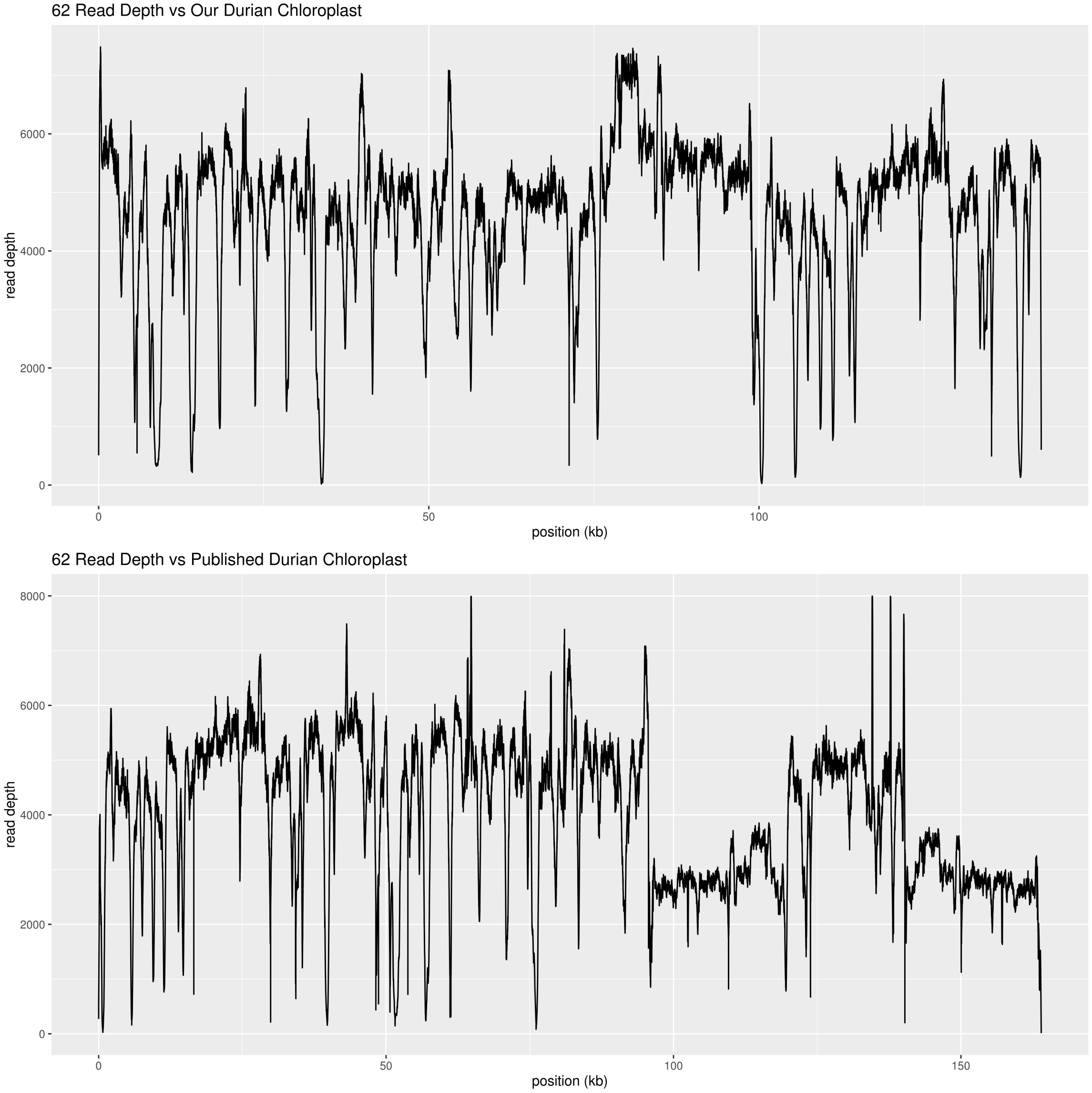

Supplementary Figure S15: Read depth of durian sample 63 Gratum Thong against our chlorplast and the published chloroplast

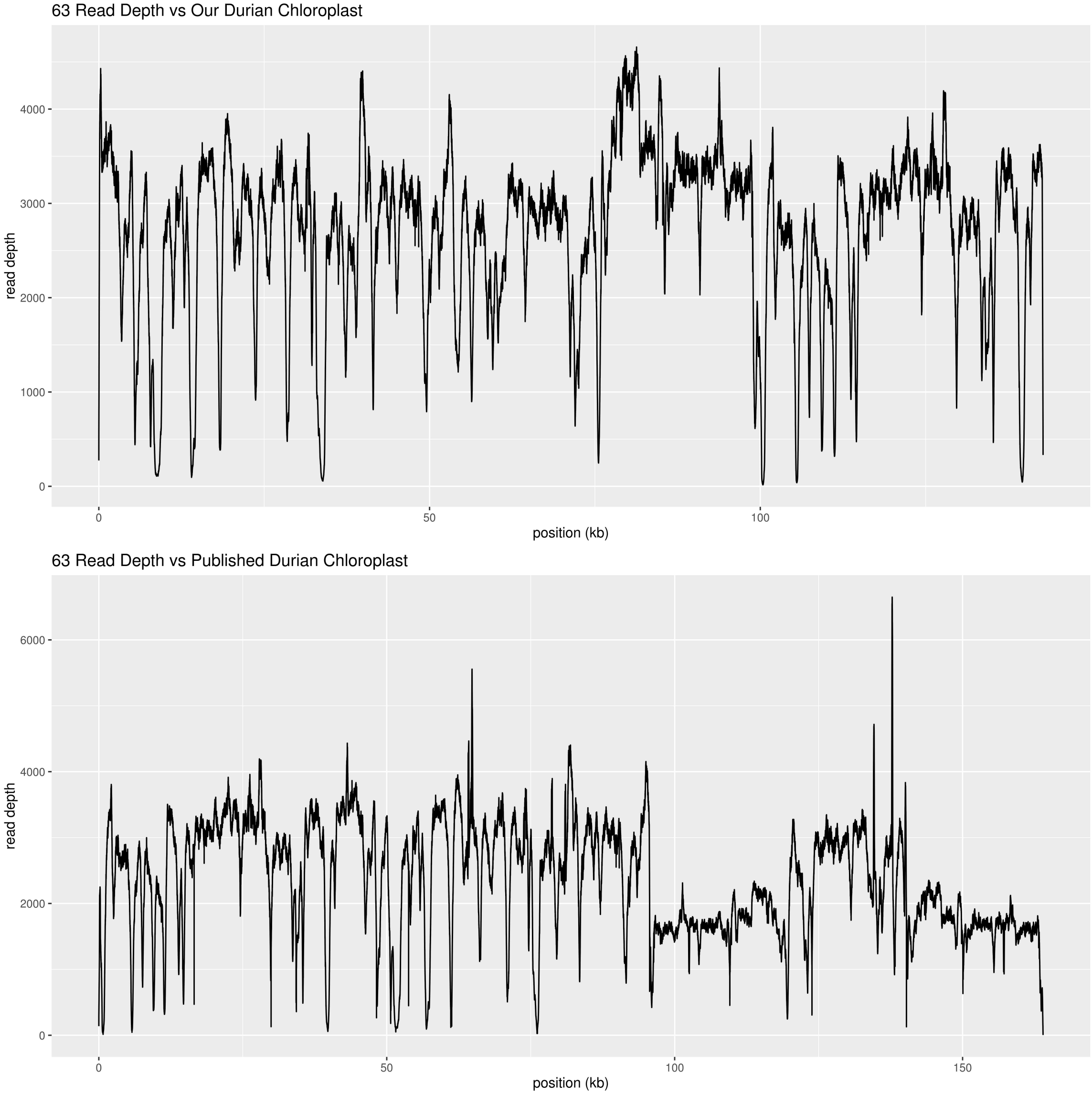

Supplementary Figure S16: Read depth of durian sample 64 Phuang Mani against our chlorplast and the published chloroplast

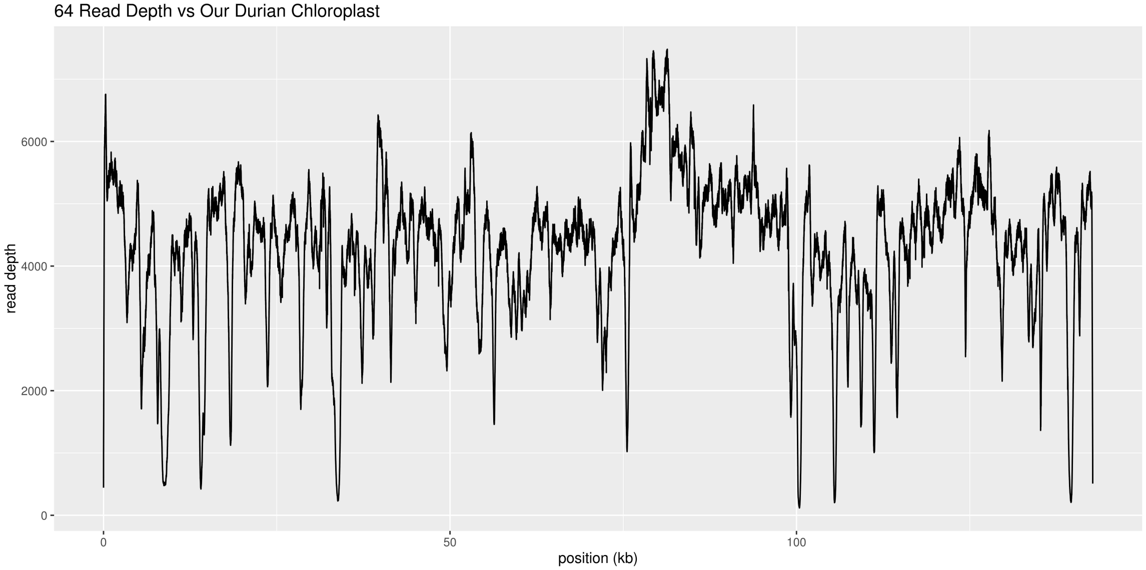

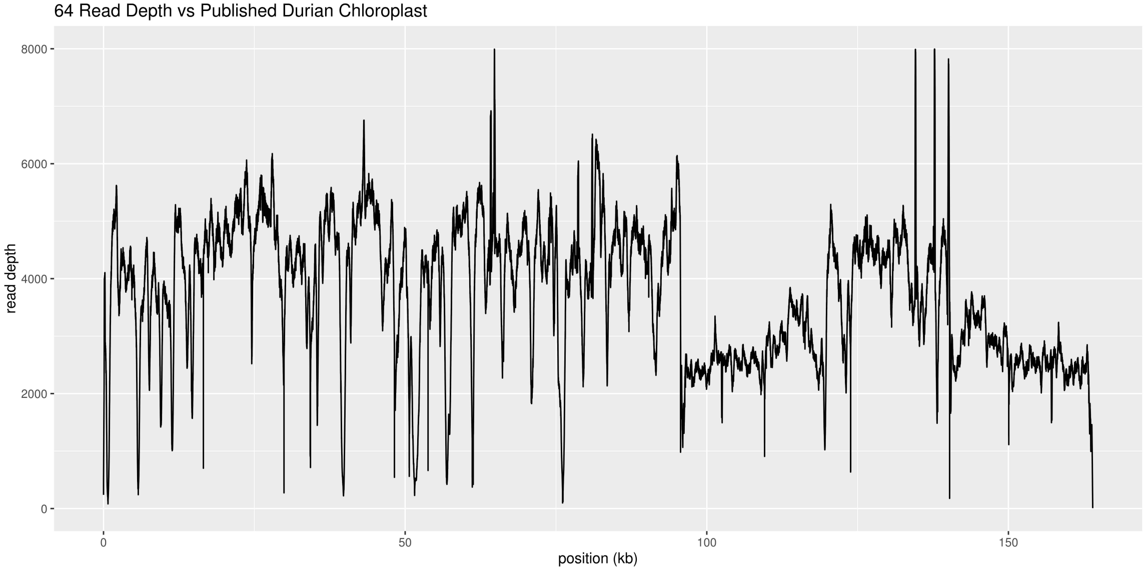

Supplementary Figure S17: Read depth of durian sample 73 Kop Thakham against our chlorplast and the published chloroplast

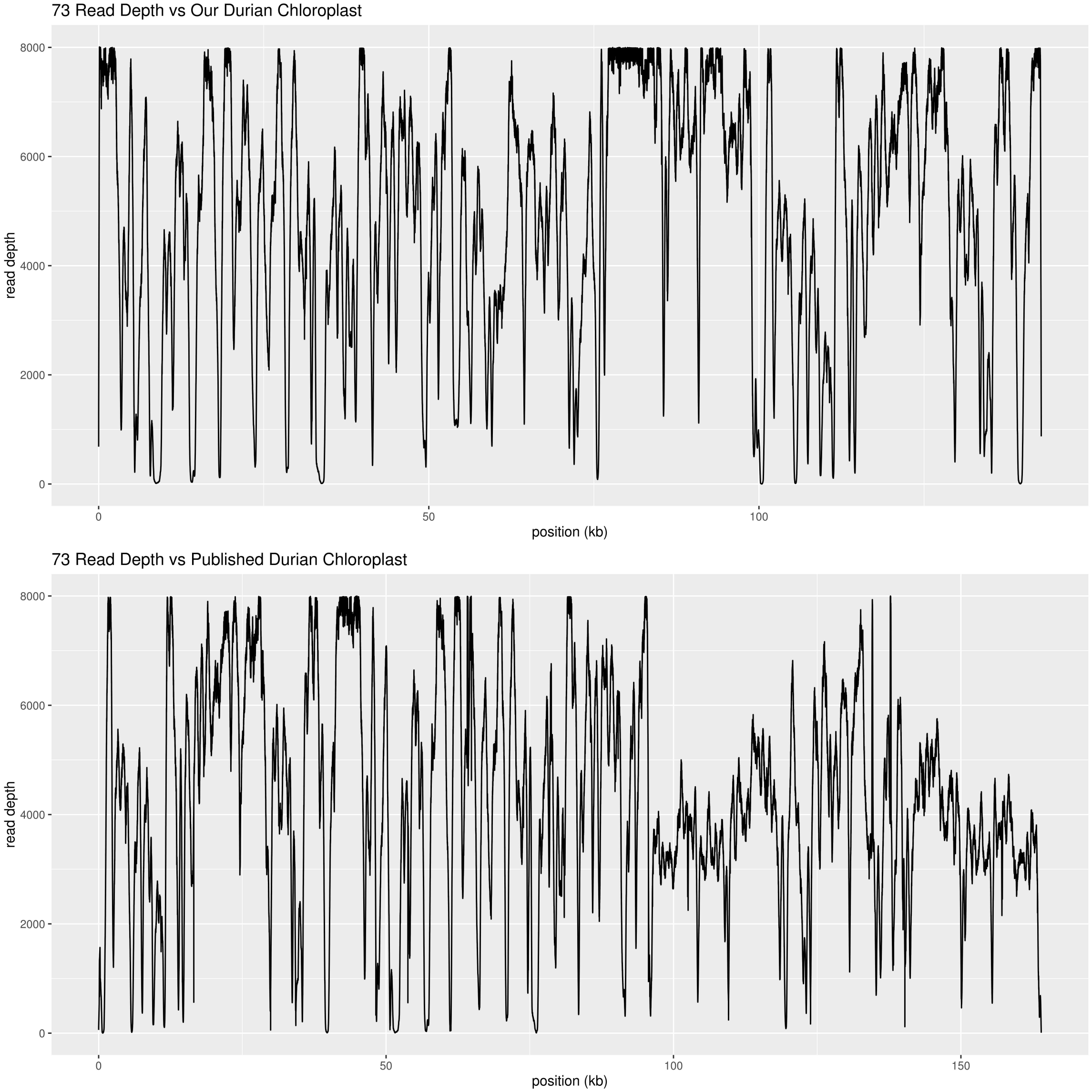

Supplementary Figure S18: Read depth of durian sample 74 Kop Thatuam against our chlorplast and the published chloroplast

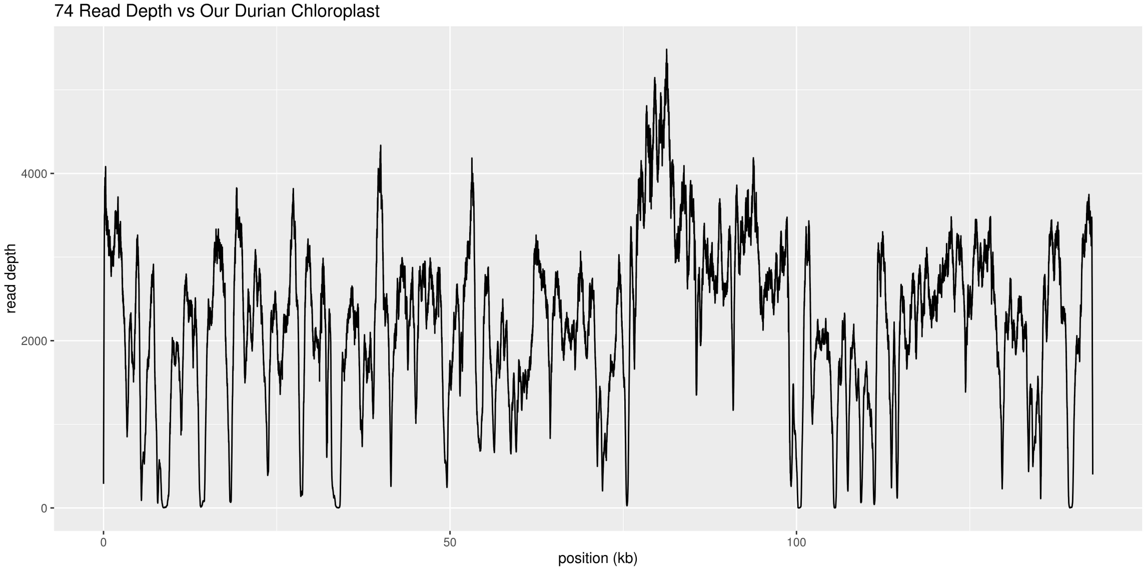

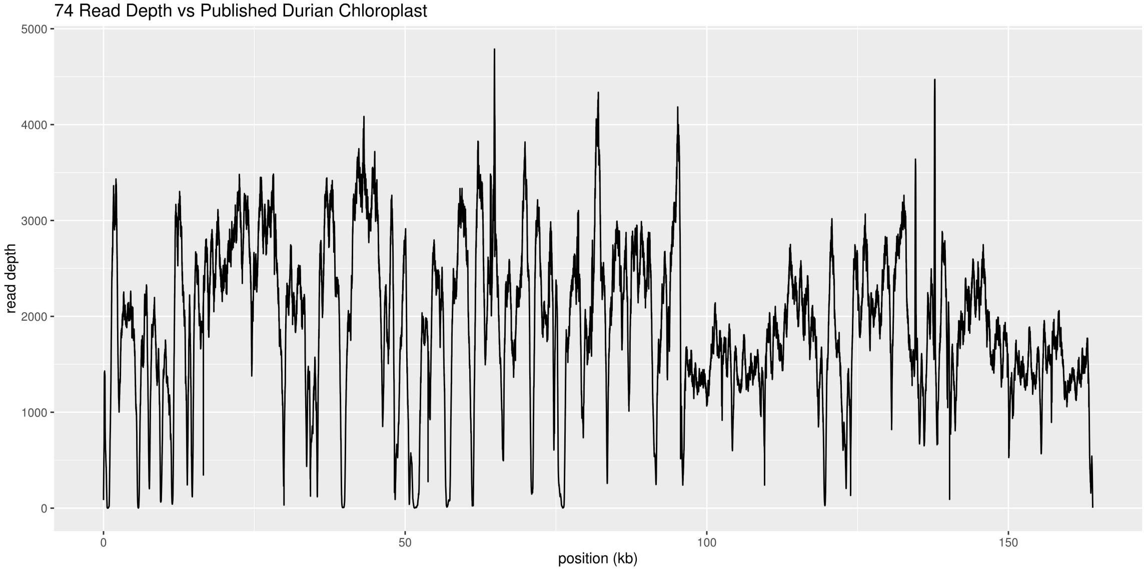

Supplementary Figure S19: Read depth of durian sample 75 Kop Maethao against our chlorplast and the published chloroplast

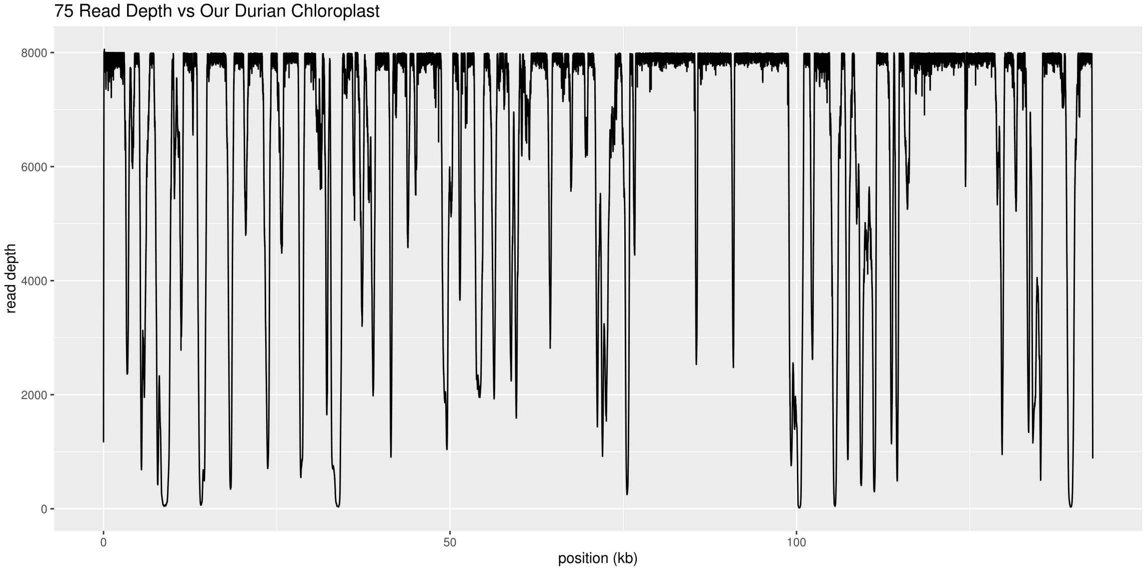

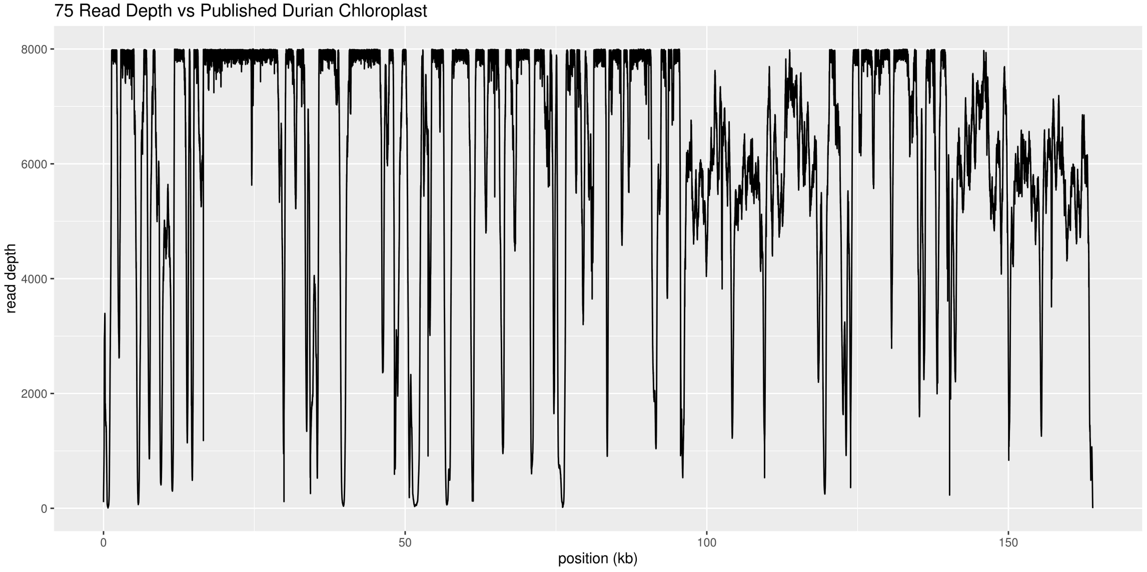

Supplementary Figure S20: Read depth of durian sample 76 Kop Suwan against our chlorplast and the published chloroplast

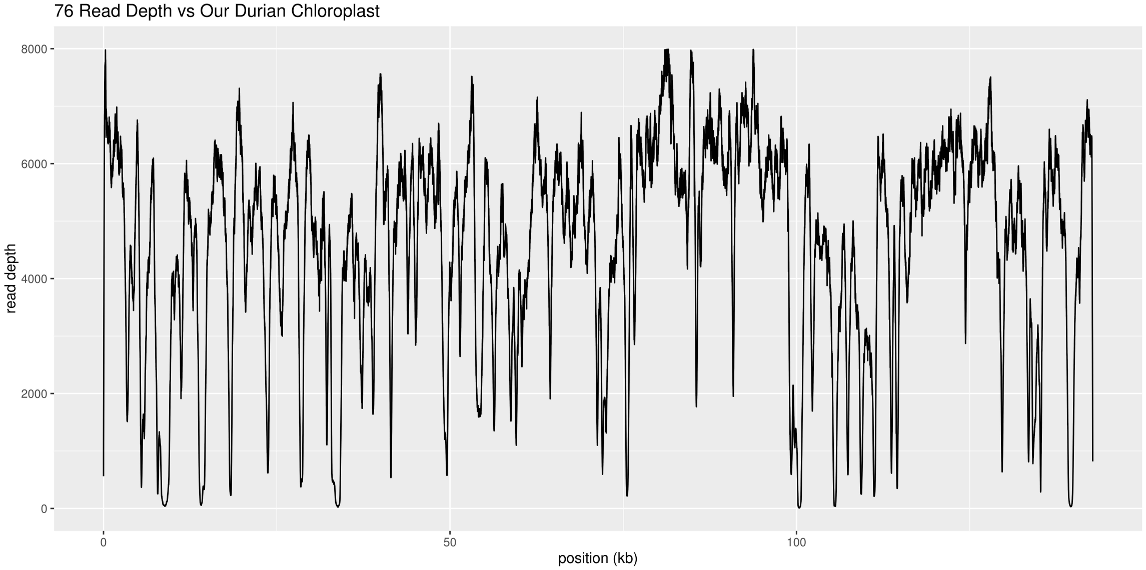

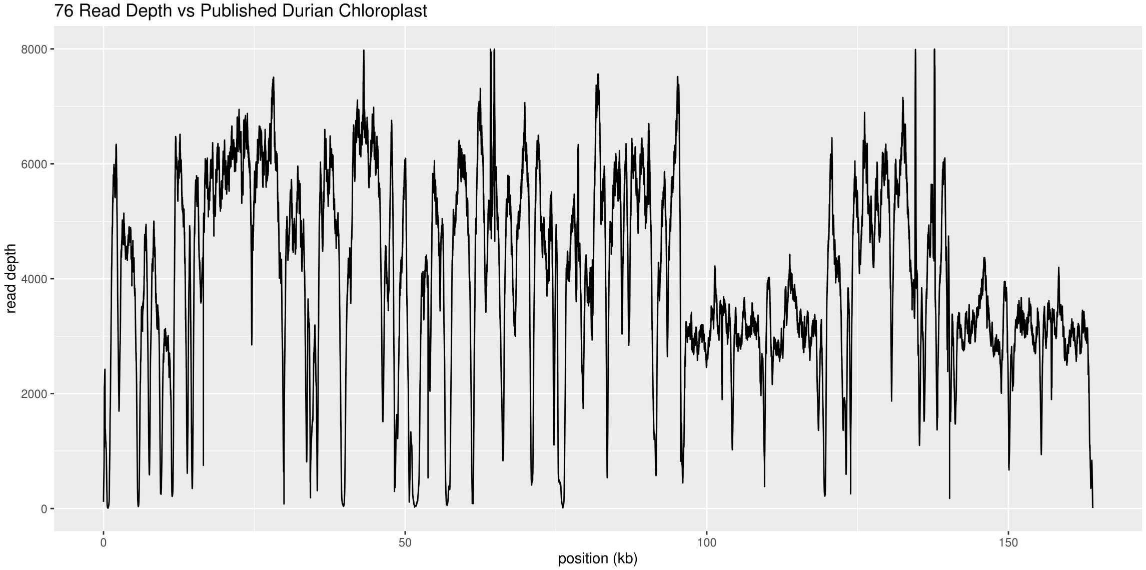

Supplementary Figure S21: Read depth of durian sample 77 Kop Watkluai against our chlorplast and the published chloroplast

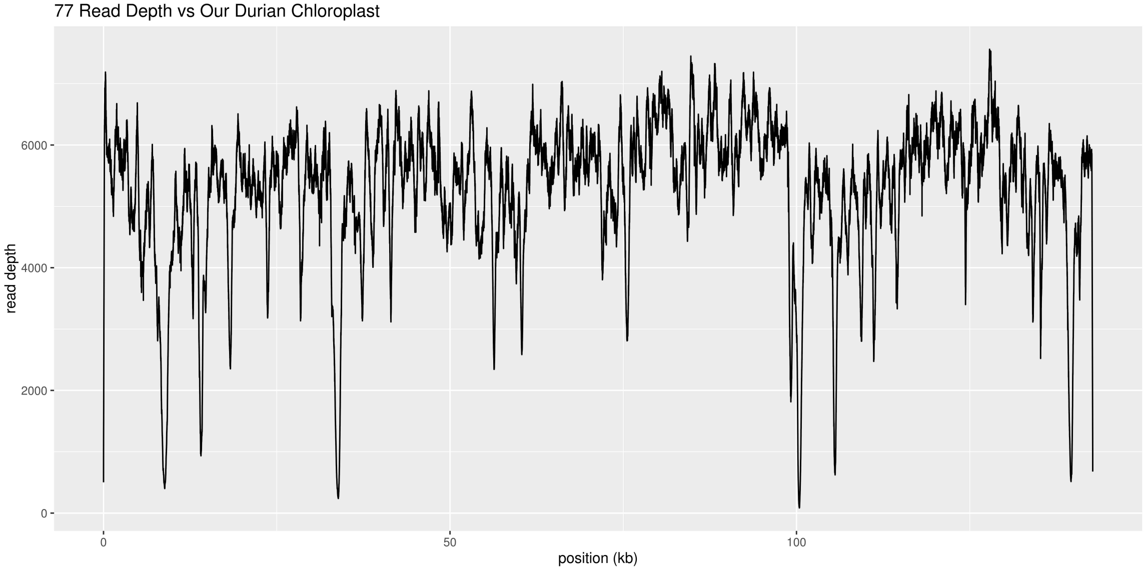

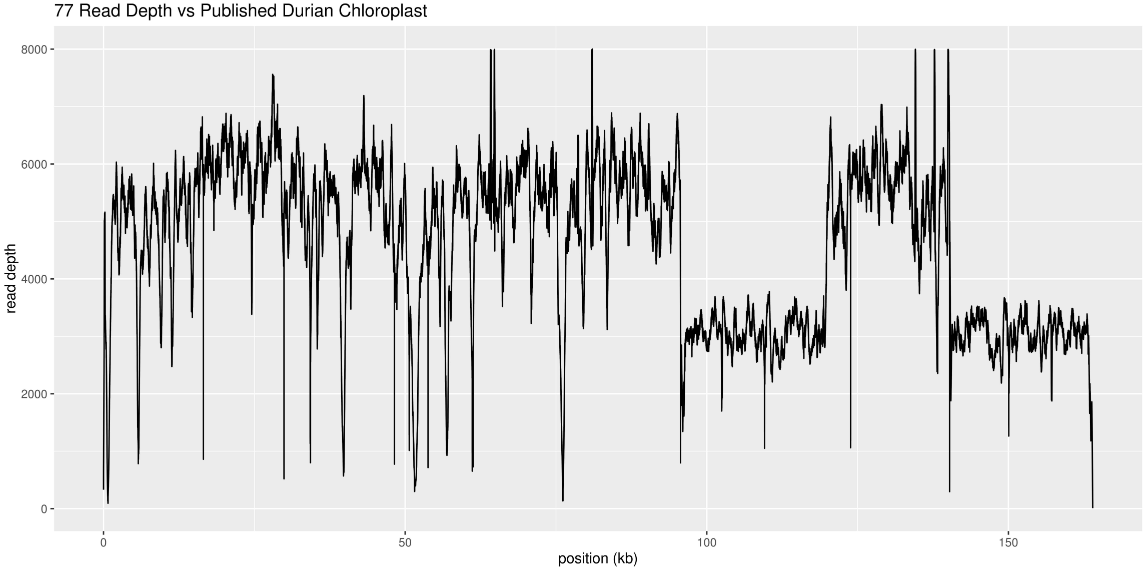

Supplementary Figure S22: Read depth of durian sample 78 Chompu Si against our chlorplast and the published chloroplast

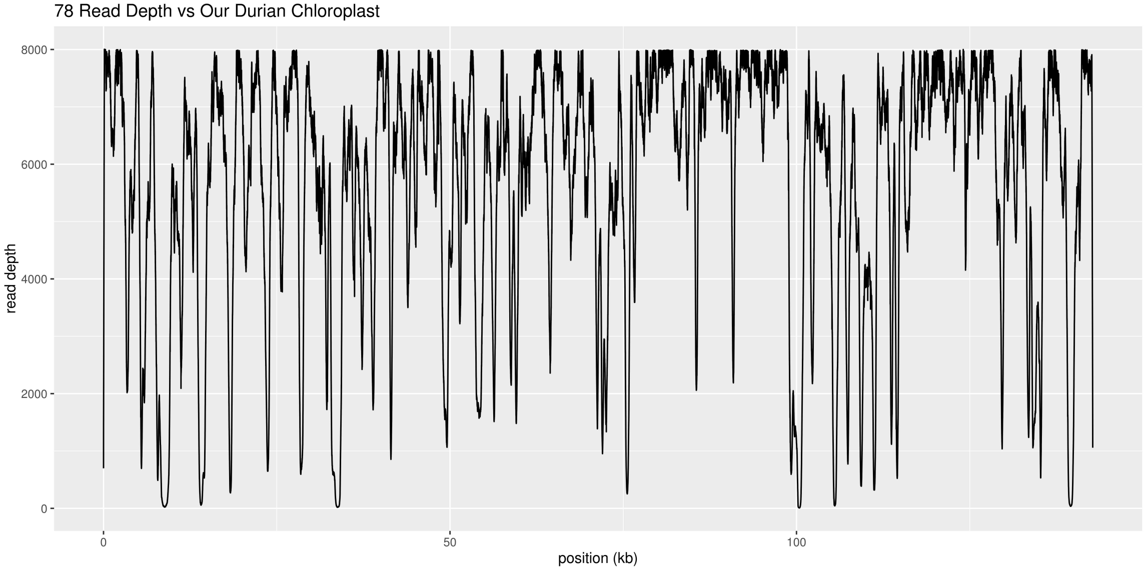

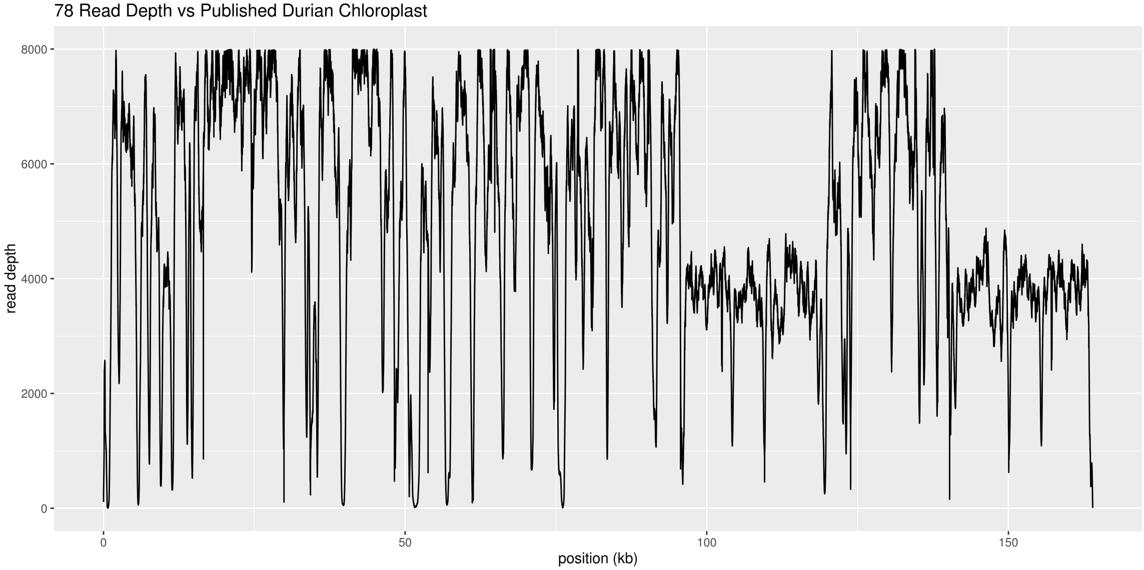

Supplementary Figure S23: Read depth of durian sample 79 Luang against our chlorplast and the published chloroplast

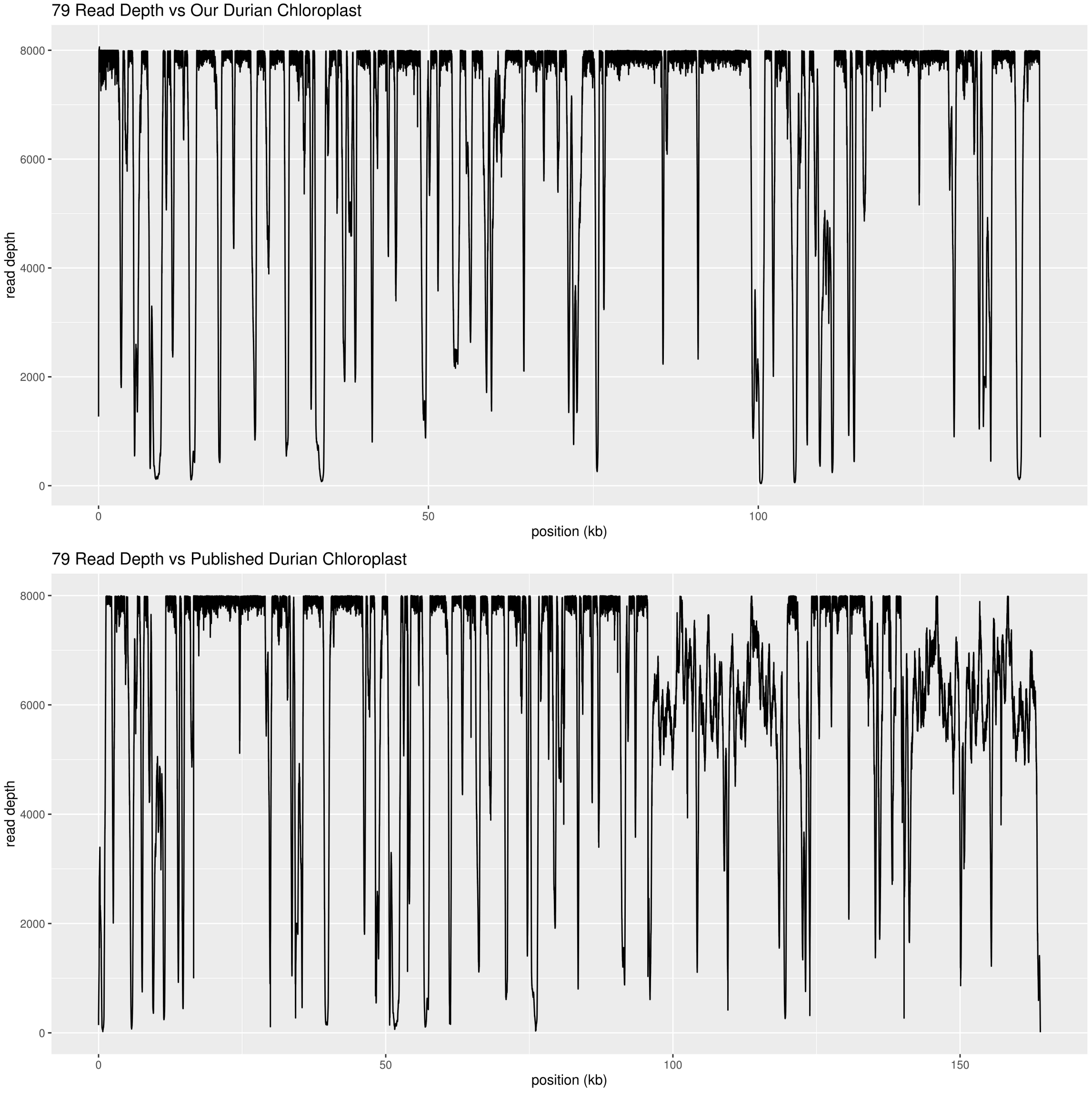

Supplementary Figure S24: Read depth of durian sample 80 Yum Mahaat against our chlorplast and the published chloroplast

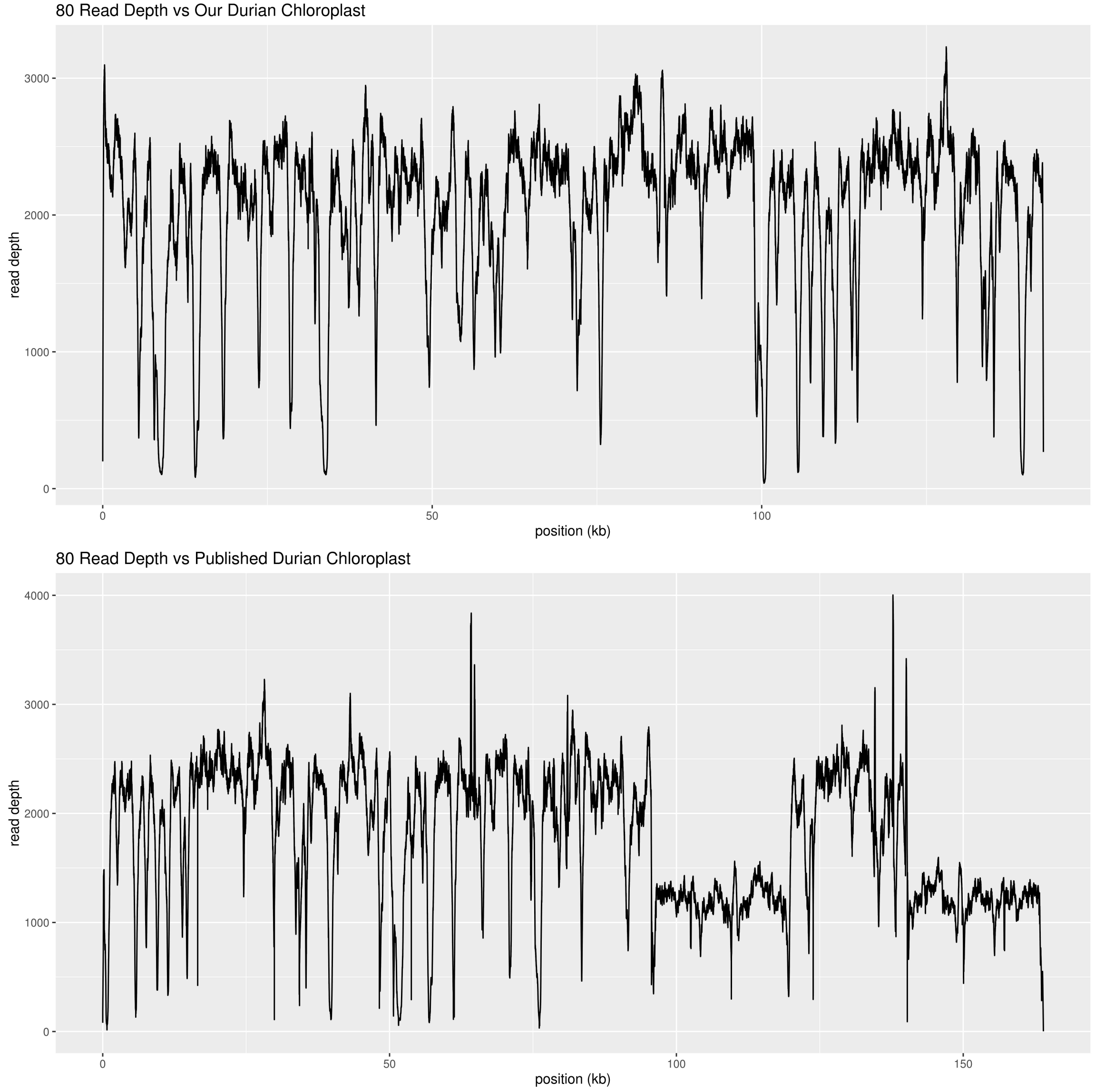

Supplementary Figure S25: Read depth of durian sample Musang King against our chlorplast and the published chloroplast with no reduction to input reads

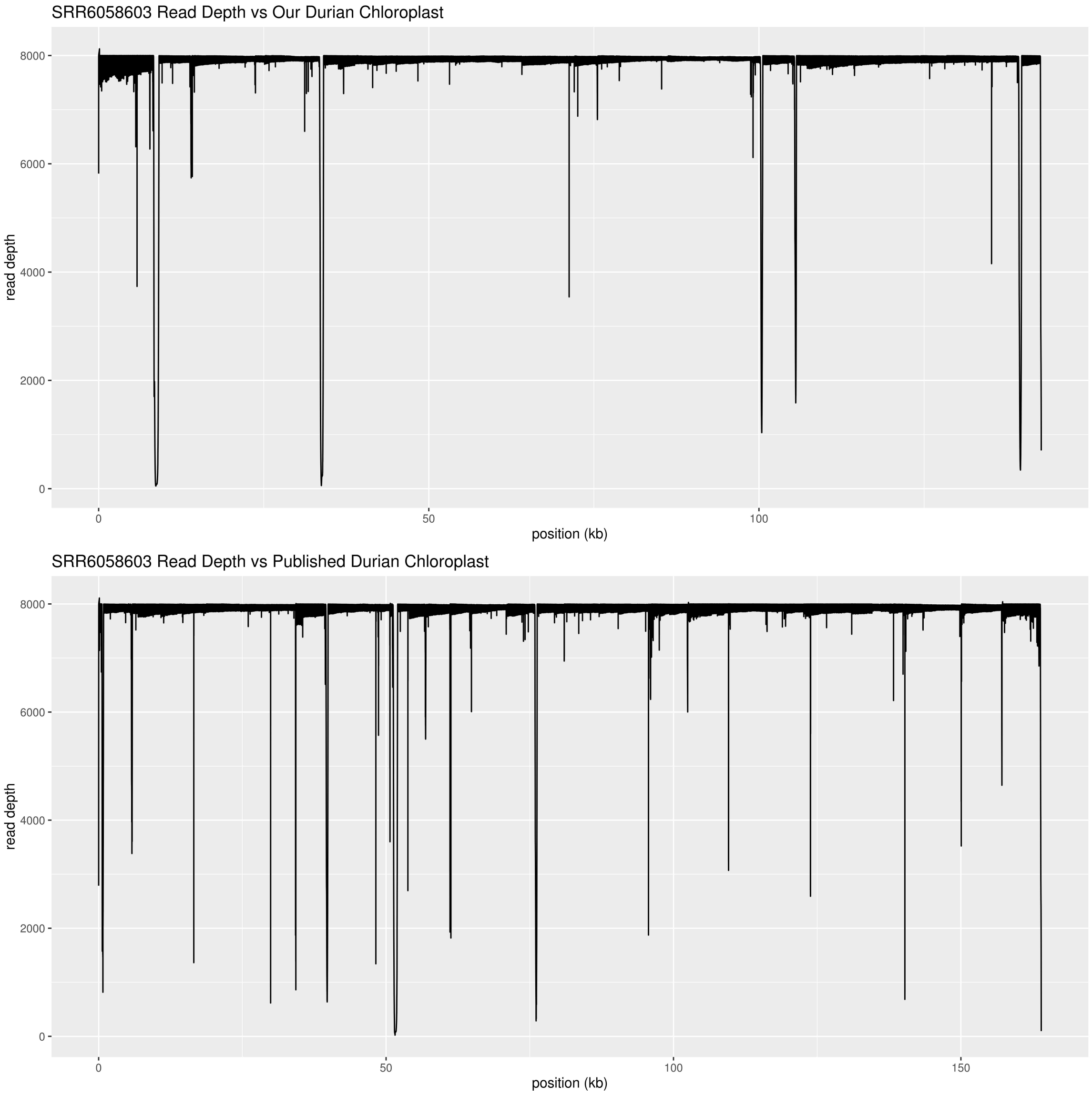

Supplement: Supplementary file 1 — Supplementary file1 [file 41598_2020_73549_MOESM1_ESM.pdf]
